# Unravelling the processes between phenotypic plasticity and population dynamics in migratory birds

**DOI:** 10.1111/1365-2656.13686

**Published:** 2022-03-23

**Authors:** Jin Liu, Weipan Lei, Xunqiang Mo, Chris J. Hassell, Zhengwang Zhang, Tim Coulson

**Affiliations:** ^1^ Key Laboratory for Biodiversity Science and Ecological Engineering, College of Life Sciences Beijing Normal University Beijing China; ^2^ Department of Zoology University of Oxford Oxford UK; ^3^ School of Geographic and Environmental Sciences Tianjin Normal University Tianjin China; ^4^ Global Flyway Network Broome WA Australia

**Keywords:** annual cycle, carrying capacity, density‐dependent, individual‐based model, loss of staging habitat, migratory birds, population dynamics, stopover duration

## Abstract

1. Populations can rapidly respond to environmental change via adaptive phenotypic plasticity, which can also modify interactions between individuals and their environment, affecting population dynamics. Bird migration is a highly plastic resource‐tracking tactic in seasonal environments. However, the link between the population dynamics of migratory birds and migration tactic plasticity is not well‐understood.
2. The quality of staging habitats affects individuals' migration timing and energy budgets in the course of migration and can consequently affect individuals' breeding and overwintering performance, and impact population dynamics. Given staging habitats being lost in many parts of the world, our goal is to investigate responses of individual migration tactics and population dynamics in the face of loss of staging habitat and to identify the key processes connecting them.
3. We started by constructing and analysing a general full‐annual‐cycle individual‐based model with a stylized migratory population to generate hypotheses on how changes in the size of staging habitat might drive changes in individual stopover duration and population dynamics. Next, through the interrogation of survey data, we tested these hypotheses by analysing population trends and stopover duration of migratory waterbirds experiencing the loss of staging habitat.
4. Our modelling exercise led to us posing the following hypotheses: the loss of staging habitat generates plasticity in migration tactics, with individuals remaining on the staging habitat for longer to obtain food due to a reduction in per capita food availability. The subsequent increasing population density on the staging habitat has knock‐on effects on population dynamics in the breeding and overwintering stage. Our empirical results were consistent with the modelling predictions.
5. Our results demonstrate how environmental change that impacts one energetically costly life‐history stage in migratory birds can have population dynamic impacts across the entire annual cycle via phenotypic plasticity.

## Graphical abstract

This work highlights the critical role of the stopover stage of northward migration in the influence of migration tactics and population dynamics of migratory birds across the whole annual cycle. The authors also demonstrate the key processes linking individual migration tactics and population dynamics.

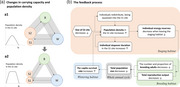

## 摘要

1. 种群因具备适应性的表型可塑性可以迅速响应环境变化，同时，表型可塑性也能够通过改变个体与其环境之间的相互作用而影响种群动态。迁徙是鸟类在季节性变化的环境中演化出的一种资源追踪型策略，其可塑性非常强。然而有关迁徙策略可塑性与候鸟种群动态之间的反馈过程尚不清楚。
2. 迁徙中停地的质量影响着候鸟个体在迁徙过程中时间与能量的权衡，进而通过影响个体在繁殖和越冬阶段的表现来影响种群动态。由于世界范围内很多迁徙鸟类的中停地都在急剧减少，因此本文希望探究在迁徙中停地丧失的情况下，鸟类的迁徙策略与种群动态之间存在何种联结。
3. 首先，我们构建了一个包含程式化迁徙种群全年周期的基于个体的模型，利用该模型提出了关于迁徙中停地的变化如何驱动候鸟个体停留时间和种群动态发生改变的假设，并通过对中国渤海湾地区迁徙中停的水鸟的调查数据的分析进行了验证。
4. 我们的假设是：迁徙中停地的丧失会导致鸟类迁徙策略的改变，由于候鸟个体在中停地可获得的食物资源减少，它们会延长在中停地的停留时间以补充能量，由此引发中停地鸟类种群密度的增长，从而对整个生活史周期内候鸟繁殖和越冬阶段的种群动态产生了连锁反应。我们的实证研究结果与建模预测一致。
5. 本研究表明，环境变化对迁徙候鸟生活史中某一个阶段的作用会通过表型可塑性对其整个周期的种群动态产生巨大影响。

## 1 INTRODUCTION

Populations can rapidly respond to environmental change via adaptive phenotypic plasticity, and this allows them to cope with profound environmental impacts (Coulson et al., 2017; Piersma & Drent, 2003; Pigliucci, 2001). Plasticity modifies interactions between individuals and their environment, ultimately affecting population dynamics (Miner et al., 2005). Migration can be an adaptive plastic tactic in seasonal environments (Lack, 1968; Newton, 2007) that allows individuals to increase reproductive output by avoiding unsuitable ecological conditions (Hedenström, 2008; Winkler et al., 2014). Plasticity of migration tactics enables migratory species to respond to environmental changes in multiple ways, such as by altering migratory routes (Dolman & Sutherland, 1995; Sutherland & Crockford, 1993), timing of migration (Balbontín et al., 2009; Gienapp et al., 2007) and through diet (Parrish, 2000). However, the link between the population dynamics of migratory species and migration tactics plasticity is not well‐understood.

Bird migration is a resource‐tracking tactic that aims to optimize a bird's energy budget in the face of fluctuating resources in seasonal environments and in the face of strong competition (Alerstam et al., 2003; Cox, 1968; Somveille et al., 2018; Winger et al., 2019). Migration is energetically costly, so birds build up fat reserves. However, carrying a large energy reserve increases flight costs and can also attract predators (Alerstam & Lindström, 1990). One tactic to minimize such costs is to stop over several times during the journeys between breeding and wintering sites to refuel (Piersma, 1988). For individuals to remain in favourable environments across their migration route, they must carefully manage the timing of departure and arrival (Alerstam et al., 2003; Alerstam & Lindström, 1990; Winkler et al., 2014). In general, individuals that arrive at breeding grounds earlier have higher reproductive success than those that arrive later (Marra et al., 1998; Norris et al., 2004), and selection favours individuals that minimize the time spent travelling during the northward migration (Lindstrom & Alerstam, 1992). Migratory birds usually spend much longer accumulating energy reserves in staging areas than in flying (Hedenström & Alerstam, 1997). Therefore, the total time spent on migration is consequently strongly influenced by the quality of, and an individual's behaviour at, staging areas (Erni et al., 2002; Hedenström & Alerstam, 1997).

For migratory species, all stages of the annual cycle are closely linked at both the individual and population levels, through carry‐over and density‐dependent effects (Harrison et al., 2011; Newton, 2007). The individual state in one stage can influence individual performance in subsequent stages, and the change in population size in one stage can influence per capita rates and consequently regulate population size in later stages (Ratikainen et al., 2008; Ryan Norris & Marra, 2007; Studds & Marra, 2005). The tactic an individual follows while at the staging area can consequently affect breeding and overwintering performance, and impact population dynamics.

Staging habitat for migratory waterbirds in the Yellow Sea is being lost in significant quantities, primarily due to land reclamation for infrastructure development and aquaculture (Bi et al., 2012; Murray et al., 2014; Yang et al., 2011). Although illegal hunting, human disturbance, exotic *Spartina* invasion and climate change impacting other parts of the annual cycle also threaten migratory birds along the East Asian Australasian Flyway (EAAF), the loss of staging habitat has been suggested as a primary cause of population declines (Amano et al., 2012; Ma et al., 2014; Melville et al., 2016; Studds et al., 2017). It is presumably because staging habitat in this system is the stage of the annual cycle where density dependence is strongest (Sutherland, 1996b). Habitat loss in staging habitats can reduce food resources, decrease foraging and fat accumulation rates of migrants (Baker et al., 2004; Morrision, 2006; Verkuil et al., 2012), increase competition and interference in the population and can have significant consequences for population regulation (Newton, 2007; Sutherland, 1996b). However, the way in which individuals respond to such changes, as well as the processes and mechanisms that cause population declines, are yet to be generally established. Migratory waterbirds along the EAAF provides a unique system to explore how individuals respond to changes in one life‐history stage and how this response would influence their populations.

To examine the effects of habitat loss within staging habitat on individual migration tactics and population dynamics, we use theoretical modelling to generate hypotheses that we next test with empirical data. First, we conducted an individual‐based modelling exercise, building a stylized full‐annual‐cycle model in which individuals follow the same migrating rules. The model led us to hypothesize that the loss of staging habitat generates plasticity in migration tactics, with individuals staying in the remaining staging site for longer to obtain food due to a reduction in per capita food availability. The increasing population density in the staging habitat has knock‐on effects on breeding and overwintering stages that impact the population dynamics, via impacts on survival and reproduction rates. We used this model to make predictions about changes in tactics that might influence population dynamics in the staging, breeding and overwintering grounds. We did this by examining wherein the life cycle density dependence operated most strongly. Next, using 13 years of survey data on 148 waterbird species in total, we examined whether observed empirical trends were consistent with hypotheses we generated from our individual‐based model. Our empirical analyses were consistent with the theoretical hypotheses our model suggested: we found that in the EAAF system where the total population size is declining along the whole flyway, population density increases at the remaining staging site as the size of the staging habitat decreases, with prolonged stopover duration for individuals. We conclude that environmental change effects on one life‐history stage in migratory birds can consequently have population dynamic impacts across the entire annual cycle via phenotypic plasticity.

## 2 MATERIALS AND METHODS

### 2.1 The individual‐based model

The individual‐based model (IBM) we constructed is a stylized model. The basic assumptions of our model are as follows: (a) the stages of the annual cycle in our model includes northward migration, staging in the course of northward migration, breeding, southward migration and overwintering; (b) all individuals followed the same set of rules in our models; (c) individuals who meet the condition for reproduction produce once each year; and (d) males are not limiting and can be ignored such that we construct a female‐only model.

#### 2.1.1 Model description

**FIGURE 1:**
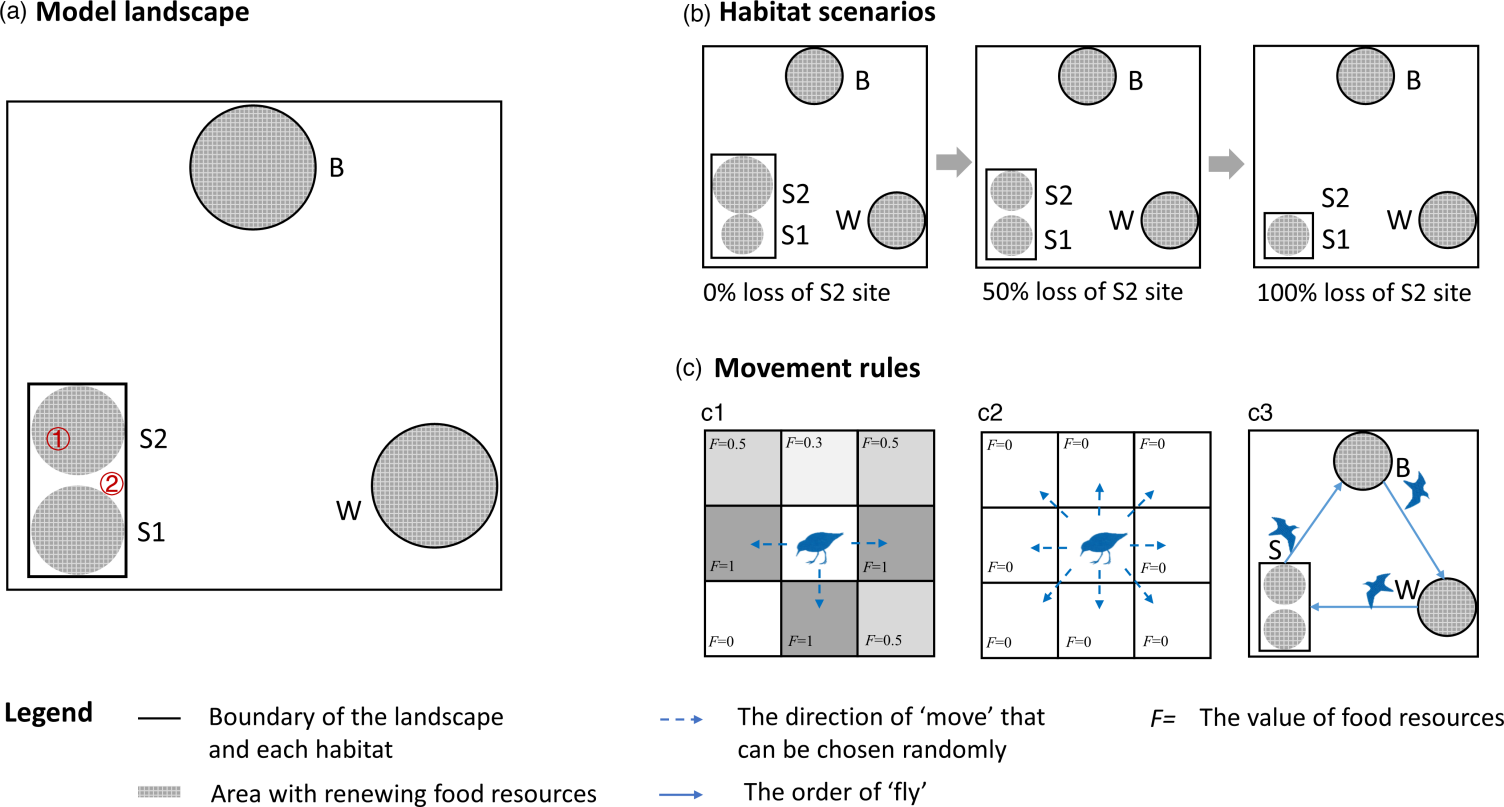
A schematic diagram of the landscape and movement rules of the individual‐based model. (a) Model landscape. ① represents the case in which food is available in the neighbouring grid cells for individuals, and ② represents the case in which food is unavailable in the neighbouring cells. The movement rules of individuals for ① and ② are shown in (c1) and (c2) respectively. (b) Three selected scenarios for the landscape in which the size of the S2 site differed. (c) Movement rules, (c1) ‘move’ under the case of ①, (c2) ‘move’ under the case of ② and (c3) the order of ‘fly’

Our IBMs include three types of habitats in the model landscape, which are wintering habitat (W), breeding habitat (B) and staging habitat (S). To examine the role of the remaining staging site on individual migratory tactics and population dynamics, we specified two alternative sites in the staging habitat—the S1 and S2 sites (Figure 1a). The size of the S1 site remained constant across all simulations, while the habitat size of the S2 site can be adjusted (Figure 1b). The total staging habitat was defined as the smallest rectangle that could encompass S1 and S2 sites, the size of the rectangle has no impact on model results, it is simply a boundary that allows individuals to move between S1 and S2 sites easily. The rest of the grid cells in the landscape are non‐habitat, where individuals pass by during migration but do not stop. Each grid cell within W, B, S1 and S2 sites contained renewing food resources, while the rest of the landscape did not include any food resources. Food resources at each grid cell renewed each time step at the habitat‐specified food recovery rate after consumption. Each time step in this model represented 1 day such that 1 year was comprised of 365 steps.

We only considered the female component of the population and characterized each individual by identity, age, reproduction status and energy reserves. The behaviours of each individual in the model included: fly, move, search for food, eat, orient, mature, reproduce and die. ‘Fly’ was the movement across habitats during the course of migration, the speed of ‘fly’ was three grid cells per time step. ‘Move’ was the movement within each habitat during the course of overwintering, staging and breeding, when individuals reached the boundary of each habitat, the behaviour of ‘move’ started, the speed of ‘move’ was one grid cell per time step. Either type of movement consumes part of an individual's energy reserves at each time step. Birds searched for food by following a random movement rule. They compared the food value on their neighbouring eight grid cells to the current one, if there was a highest food value, birds moved towards this cell; if food values were the same on more than one cell, birds randomly chose a direction to move (Figure 1c1). Birds ate and increased their energy reserves when food was available; if food was unavailable, then birds randomly moved to a neighbouring cell and did not eat (Figure 1c2). There was no randomness of food acquisition; as long as food resources were available, the behaviour of ‘eat’ happened and energy was stored. When the energy reserve of an individual reached the energy threshold for departure, or the time reached for the latest possible departure arrived, the individual first oriented, adjusting its facing to the centre of the next destination habitat in the next time step, then flew towards it. Individuals whose energy reserves reached the threshold for reproduction matured and reproduced once each year. Reproduction also consumed individuals' energy reserves. Hatchlings were set with an initial value of age, reproduction status and energy reserves. Individuals aged 15 years, or with zero energy reserves, died and were removed from the population. Details of events and decisions of the models are provided in Figure S1 and Appendix S2.

The energy reserves of each individual were assumed to be dependent on their initial energy, energy gained and energy expended. The expected energy gained from food relied on both population density and food density (Goss‐Custard et al., 2002). The stopover duration of a bird in the staging habitat was related to the energy requirement for migration and the rate of energy acquisition (Hedenström & Alerstam, 1997). Parameter values in this model were consensus values, drawn from empirical studies of multiple migratory waterbirds (Table S1). These values were not generated from statistical analyses of individual data, due to the lack of available data. The calculation of individual energy reserves and stopover duration is provided in Appendix S2.

#### 2.1.2 Model implementation

Our main goal is to examine the effects of habitat change in the staging habitat on individual stopover duration in the course of northward migration and population dynamics across the entire life cycle and to identify the processes that connect them. To further examine whether our hypothesized processes only occur when the staging habitat became the stage with the lowest carrying capacity during the annual cycle, rather than the breeding or wintering stage, we tested impacts on individual stopover duration and population dynamics by reducing carrying capacity at other life cycle stages (Table S2 in Appendix S1 and Appendix S3). In addition, since individuals with different breeding tactics (capital breeding and income breeding) have different energy budgets along the life cycle, we also tested the effects of breeding tactics on model outputs (Table S2 in Appendix S1 and Appendix S3).

The average daily population density and the total number of individuals, individual stopover duration, individual energy reserves and energy accumulation rate during stopover were recorded at equilibrium and were examined by comparing the mean value of simulation results. The model was run 10 times for each scenario and for 30 years in each simulation, which was sufficient to converge to stationary dynamics. Results were obtained from year 5 to year 30 of the simulation (Figure S2).

The average daily population density was recorded at the S1 site; it was the mean of the abundance per day at the S1 site during the period of the stopover stage each year. The total number of individuals for the population and for the three types of habitats were recorded each year respectively. Individuals were recorded in age classes and reproduction status (juveniles, breeding adults and non‐breeding adults).

The per capita reproduction rate for adults, the reproduction rate among breeding adults and the survival rate were all calculated. The per capita reproduction rate was the ratio of the number of juveniles and the number of all adults in the breeding stage; the reproduction rate among breeding adults was the ratio of the number of juveniles and the number of breeding adults; survival rate was split into two periods—one is the survival rate of the northward migration (the ratio between the number of adults at the breeding habitat and the number of individuals on the last day of wintering stage), and the other is the survival rate of the southward migration (the ratio between the number of individuals at the wintering habitat and the number of individuals at the breeding habitat).

Mean individual stopover duration in the S1 site was recorded each year. Individual departure energy reserves were recorded for three types of habitats respectively, as the energy reserves at the last day before the individual left the habitat range. Individual energy reserves during the stopover period were recorded at each time step when the individual stayed in the staging habitat. The energy accumulation rate during stopover was calculated by dividing energy gained from the staging habitat by stopover duration.

#### 2.1.3 Sensitivity analysis

To examine the impacts of parameter values on the model outputs and to test the robustness of the model results were to variation in parameter values, we conducted a local sensitivity analysis (see Methods and Results in Appendix S4).

### 2.2 Empirical data

Our empirical study was conducted in the wetlands in the north of Bohai Bay, between 38°36′‐39°13′N and 117°11′‐118.22′E, located in the northwest of the Yellow Sea (Figure S3). Despite habitat being lost in the surveyed area, the loss of favoured habitat was minimal, with the highest food density for migratory waterbirds compared to other staging sites in the Northern Yellow Sea (Peng et al., 2021; Wang et al., 2021; Yang et al., 2016). Our surveyed area is most equivalent to the S1 site in our IBMs. Survey data of migratory waterbirds were collected at boreal spring between 2004 and 2018 (details of the study area and data collection are provided in Appendix S5). All surveys were carried out under permits from Tianjin Municipal Bureau of Planning and Natural Resources and Luannan Forestry Bureau. This study did not require ethical approval.

#### 2.2.1 Statistical analyses for bird number trends

The abundance of all waterbirds and the abundance of the most common species were analysed in this study. Survey sites that were surveyed on less than 30% of survey dates were excluded from the analysis. To estimate total waterbird abundance, we summed the counts of all species *i* (including unidentified species) observed from all of the survey sites *k* in each day *j*, denoting it as *N*_*a*_. To identify the most common species, we used two methods, the ‘Frequency‐based Method’ and the ‘Distribution‐based Method’, to select 25 common species (S5). The number of these 25 species observed on each day *j* and survey site *k* was denoted as *N*_*b*_.

We calculated survey effort as the number of observers each day at each site as *E*_*j*,*k*_. We transformed date to Julian date *t*, and calculated *t* ^2^, because the temporal changes in bird abundance at staging habitat during the period of stopover is often quadratic (Thompson, 1993), and visual examination of our data also revealed a quadratic relationship. We treated ‘year’ (*y*) as a continuous variable in our models, and we treated ‘survey site’ (*k*) as a categorical variable.

Waterbird abundance (including *N*_*a*_, *N*_*b*_) was used as response variables, and *E*_*j*,*k*_, *k*, *t*, *t* ^2^, *i* and *y* were used as explanatory variables. The distribution of *N*_*a*_ and *N*_*b*_ was well‐described as an overdispersed Poisson (variance greater than mean), so we fitted GLM with a ‘quasi‐Poisson’ error structure in program R. The regression equations were of the form:

(1) $$\begin{array}{c} N_{a}=\exp(\alpha_{1}+\beta_{11}\left(y\right)+\beta_{12}\left(E_{j,k}\right)+\beta_{13}\left(y\times E_{j,k}\right)+\beta_{14}\left(k\right)\\ \;+\beta_{15}\left(y\times k\right)+\beta_{16}\left(t\right)+\beta_{17}\left(t^{2}\right)+\beta_{18}\left(y\times t\right)+\beta_{19}\left(y\times t^{2}\right)+\varepsilon_{1}), \end{array}$$

(2) $$\begin{array}{c} N_{b}=\exp\;(\alpha_{2}+\beta_{21}\;\left(y\right)+\beta_{22}\;\left(E_{j,k}\right)+\beta_{23}\;\left(y\times E_{j,k}\right)+\beta_{24}\left(k\right)\\ \;+\beta_{25}\;\left(y\times k\right)+\beta_{26}\;\left(t\right)+\beta_{27}\;\left(t^{2}\right)+\beta_{28}\;\left(y\times t\right)\\ \;+\beta_{29}\;\left(y\times t^{2}\right)+\beta_{210}\;\left(i\right)+\beta_{211}\;\left(y\times i\right)+{ɛ}_{2}) \end{array}$$

#### 2.2.2 Statistical analyses for stopover duration trends

We estimated the stopover duration for each common species by using our survey data. Since our survey data revealed a quadratic relationship between Julian date and bird abundance (Figure S4a), we first estimated normal distributions of bird abundance within the period of stopover for each species each year (Figure S4b). To do this, we extracted the mean date and variance in date for each species each year from the survey data, and scaled the curves by the bird number from the survey data. Then we estimated the date by which each quantile of the distribution of bird abundance is reached, date at 2.5% quantile and 97.5% quantile was the date of arrival at staging habitat and the date of departure from staging habitat for each species each year within 95% confidential interval respectively (Figure S4c). We calculated the stopover duration based on the arrival date and departure date for each species each year, denoting it as *T*_*i*,*y*_ (details are provided in Appendix S5.4).

The distribution of *T*_*i*,*y*_ was normal distribution, so we fitted linear model in program R to test the stopover duration trends across years for each common species, the regression equation was of the form:

(3) $$T_{i,y}=\alpha_{3}+\beta_{31}\left(y\right)+\beta_{32}\left(i\right)+\beta_{33}\left(y\times i\right)+\varepsilon_{3}.$$

The correlation between stopover duration and bird abundance.

We fitted a regression between bird abundance and stopover duration to test the correlation between them. We calculated the mean abundance during the stopover period for each species *i* each year *y*, denoting it as *N*_*c*_. *N*_*c*_ was used as the response variable, and *y*, *i* and *T*_*i*,*y*_ were used as explanatory variables. The distribution of *N*_*c*_ was an overdispersed Poisson, so we fitted GLM with a ‘quasi‐Poisson’ error structure in program R. The regression equations were of the form:

(4) $$\begin{array}{c} N_{c}=\exp(\alpha_{4}+\beta_{41}\left(y\right)+\beta_{42}\left(i\right)+\beta_{43}\left(y\times i\right)+\beta_{44}\left(T_{i,y}\right)\\ \;+\beta_{45}\left(y\times T_{i,y}\right)+\beta_{46}\left(i\times T_{i,y}\right)+\varepsilon_{4}). \end{array}$$

We used the ANOVA command in R to assess the significance of each variable, used adjusted *R* ^2^ to assess the goodness‐of‐fit of the linear model, and used 1 − (residual deviance/null deviance) to assess the goodness‐of‐fit of the GLMs.

## 3 RESULTS

### 3.1 IBM: Population dynamics and individual stopover duration at the staging habitat

**FIGURE 2:**
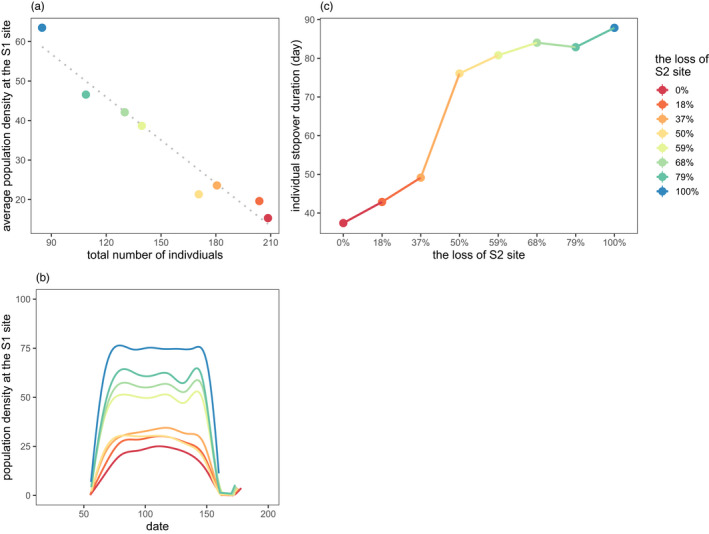
Population dynamics and stopover duration as the size of the staging habitat decreased across the eight scenarios. (a) The relationship between average daily population density at the S1 site and the total number of individuals; (b) the curves of population density at the S1 site during the period of stopover; (c) individual annual stopover duration in relation to the size of staging habitat

As the size of the S2 site decreased through eight scenarios in the model 2 simulations, there was a decrease in the total number of individuals, and an increase in average daily population density at the S1 site (Figure 2a). The curve of daily population density at the S1 site became steeper in the early and late phases of the stopover period, and higher and wider when the population reached peak numbers (Figure 2b), showing that the population took less time to reach the peak number and remained at the peak number for a longer period of time. Individual's annual stopover duration at the S1 site increased as the size of the S2 site decreased (Figure 2c). It suggests that as the habitat loss of the S2 site intensified, individuals stayed longer in the S1 site where the habitat size remains constant.

### 3.2 IBM: Individual energy reserves

**FIGURE 3:**
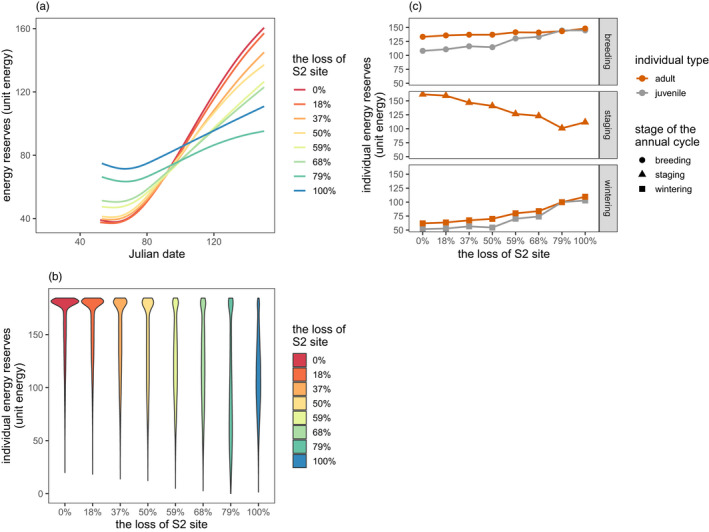
Individual energy reserves across the annual cycle. (a) The energy accumulating rate during the period of stopover when individuals were at the staging habitat. (b) The distribution of individual departure energy reserves from the staging habitat. (c) Individual departure energy reserves at each annual cycle stage

As the size of the S2 site decreased in model 2 simulations, the individual energy accumulation rate when birds remained in the staging habitat decreased (Figure 3a), and the distribution shifted towards lower energy reserves with fewer individuals having reached the energy threshold for departure from the staging habitat (Figure 3b). The energy reserves when individuals left the staging habitat were decreased; however, the departure energy reserves from the breeding habitat and wintering habitat were increased, both in adults and juveniles (Figure 3c).

### 3.3 IBM: Population structure and demography

**FIGURE 4:**
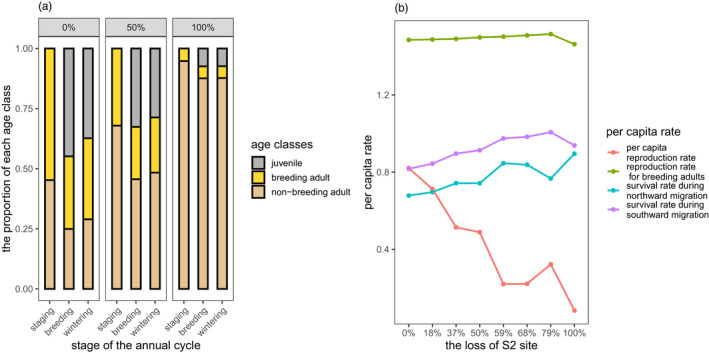
Population structure and demography as the size of the staging habitat decreased across the eight scenarios. (a) The proportion of individuals in different age classes and maturity status in three stages of the annual cycle. For illustration purposes, we only show three habitat scenarios here (0% loss of S2, 50% loss of S2 and 100% loss of S2) for ease of reading, the complete figure can be found in Figure S5. (b) Per capita rates of reproduction for all adults, reproduction for breeding adults, survival during southward migration and survival during northward migration

As the size of the S2 site decreased in model 2 simulations, the proportion of breeding adults and juveniles in the population decreased, while the proportion of non‐breeding adults increased in each life cycle stage (Figure 4a). The per capita reproduction rate decreased because of an increased proportion of non‐breeding adults, but the reproduction rate among breeding adults increased. The survival rate in both the northward migration and southward migration increased (Figure 4b).

### 3.4 Empirical data: Overall abundance

**FIGURE 5:**
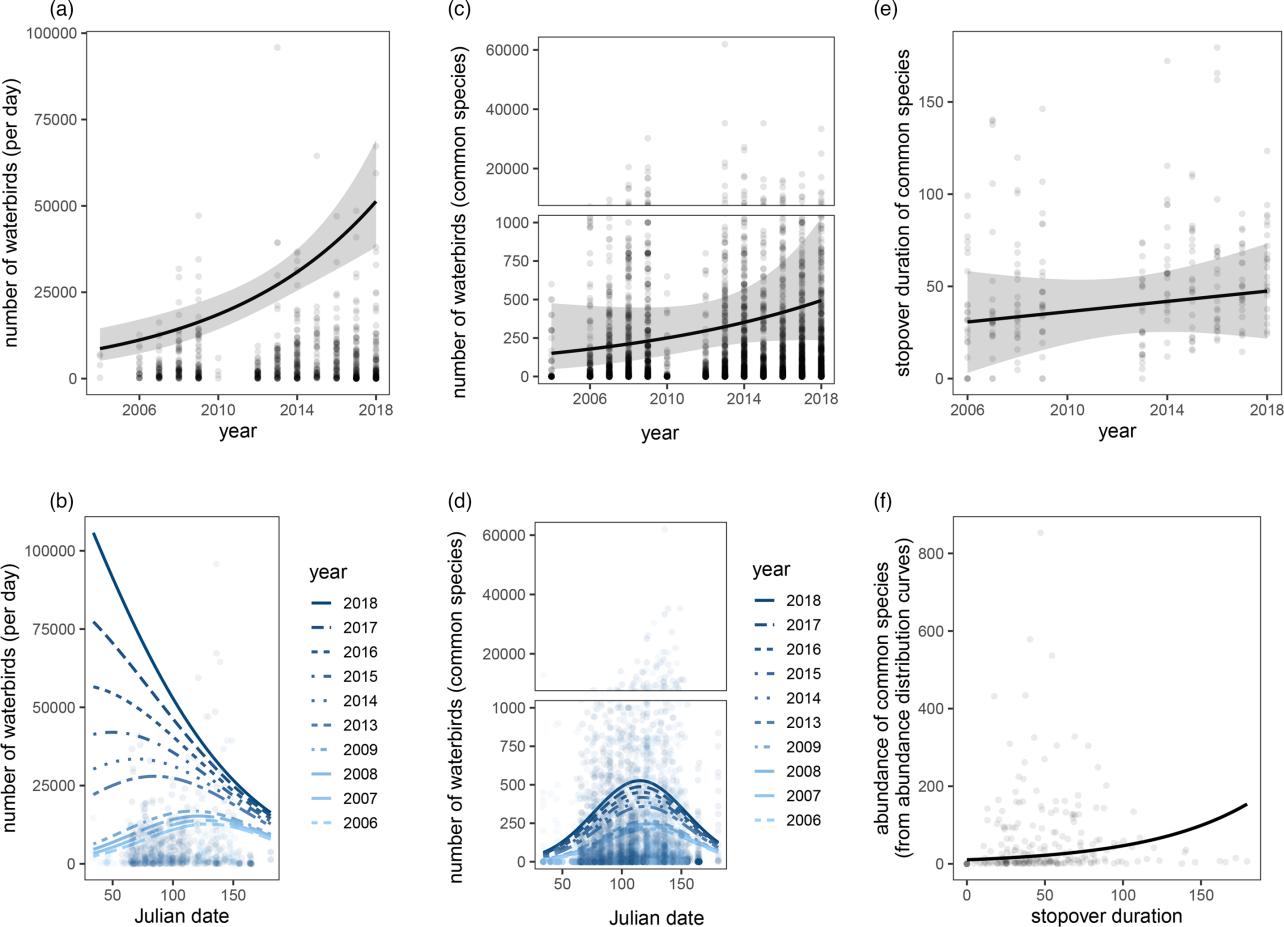
Trends in waterbird abundance and stopover duration from the survey data. (a) Overall waterbird abundance as a function of year; (b) overall waterbird abundance as a function of Julian date in different years; (c) the abundance of the most common species as a function of year; (d) the abundance of the most common species as a function of Julian date in different years; (e) stopover duration of common species as a function of year; (f) the correlation between stopover duration and the abundance of common species

The analysis of the abundance of all recorded species revealed an increase in the numbers of waterbirds at a rate of 61.85% per year (*F*_1,809_ = 13.60, *p* < 0.001) (Figure 5a). Even though effort affected abundance estimates (*F*_1,808_ = 11.04, *p* < 0.001) with more birds counted as effort increased, there was no interaction between year and effort (*F*_1,787_ = 0.094, *p* = 0.76). There was a positive relationship between waterbird abundance and Julian date (*F*_1,789_ = 5.98, *p* = 0.015). This slowed, and became negative with time, as the quadric term of date (*F*_1,788_ = 10.34, *p* = 0.0014) indicated a parabola of waterbird abundance within the migration season. There was also an interaction between year and the quadric term of date (*F*_1,768_ = 17.96, *p* < 0.001), showing that the parabola shape within the migration season changed across years (Figure 5b). The average waterbird abundance (*F*_18,790_ = 30.81, *p* < 0.001) and the annual trend (*F*_18,769_ = 3.59, *p* < 0.001) differed between survey sites (Figure S9a). The GLM explained 51.1% of the deviance.

### 3.5 Empirical data: The abundance of the most common species

In the analysis of the most common species, the abundance of common species increased at a rate of 35.13% per year (*F*_1,18,418_ = 6.92, *p* = 0.009) (Figure 5c), after correcting for effects from observers' effort (*F*_1,18,417_ = 47.63, *p* < 0.001) and the interaction between year and effort (*F*_1,18,372_ = 6.94, *p* = 0.008), survey sites (*F*_18,18,399_ = 95.28, *p* < 0.001) and the interaction between year and survey sites (*F*_18,18,354_ = 5.96, *p* < 0.001) (Figure S9b), species (*F*_24,18,373_ = 80.14, *p* < 0.001) and the interaction between year and species (*F*_24,18,328_ = 6.96, *p* < 0.001). There was a quadratic association between Julian date and the number of birds (linear term, *F*_1,18,398_ = 13.21, *p* < 0.001; quadratic term *F*_1,18,397_ = 97.79, *p* < 0.001) (Figure 5d), but the interaction between year and Julian date was not statistically significant (linear term, *F*_1,18,352_ = 0.52, *p* = 0.470; quadratic term *F*_1,18,353_ = 2.57, *p* = 0.109). The GLM explained 45.9% of the deviance.

### 3.6 Empirical data: Stopover duration

The analysis of the stopover duration of common species revealed an increasing trend at the rate of 1.39 days per year (*F*_1,245_ = 14.58, *p* < 0.001) (Figure 5e). The stopover duration (*F*_24,221_ = 7.07, *p* < 0.001) and its temporal trends (*F*_24,197_ = 1.87, *p* = 0.011) were different among species (Figure S9c). The adjust *R*‐square of the linear model was 0.423 (*F*_49,197_ = 4.67, *p* < 0.001).

In the analysis of the correlation between bird abundance and the stopover duration, abundance was positively related to stopover duration (*F*_1,220_ = 18.26, *p* < 0.001) (Figure 5f), also positively related to year (*F*_1,245_ = 56.80, *p* < 0.001). The abundance (*F*_24,221_ = 35.79, *p* < 0.001) was different across species. The relation between year and abundance (*F*_24,196_ = 3.27, *p* < 0.001) and the relation between stopover duration and abundance (*F*_24,172_ = 2.23, *p* = 0.002) were different across species. The interaction between year and stopover duration was not statistically significant (*F*_1,171_ = 0.74, *p* = 0.390). The GLM explained 85.7% of the deviance.

## 4 DISCUSSION

**FIGURE 6:**
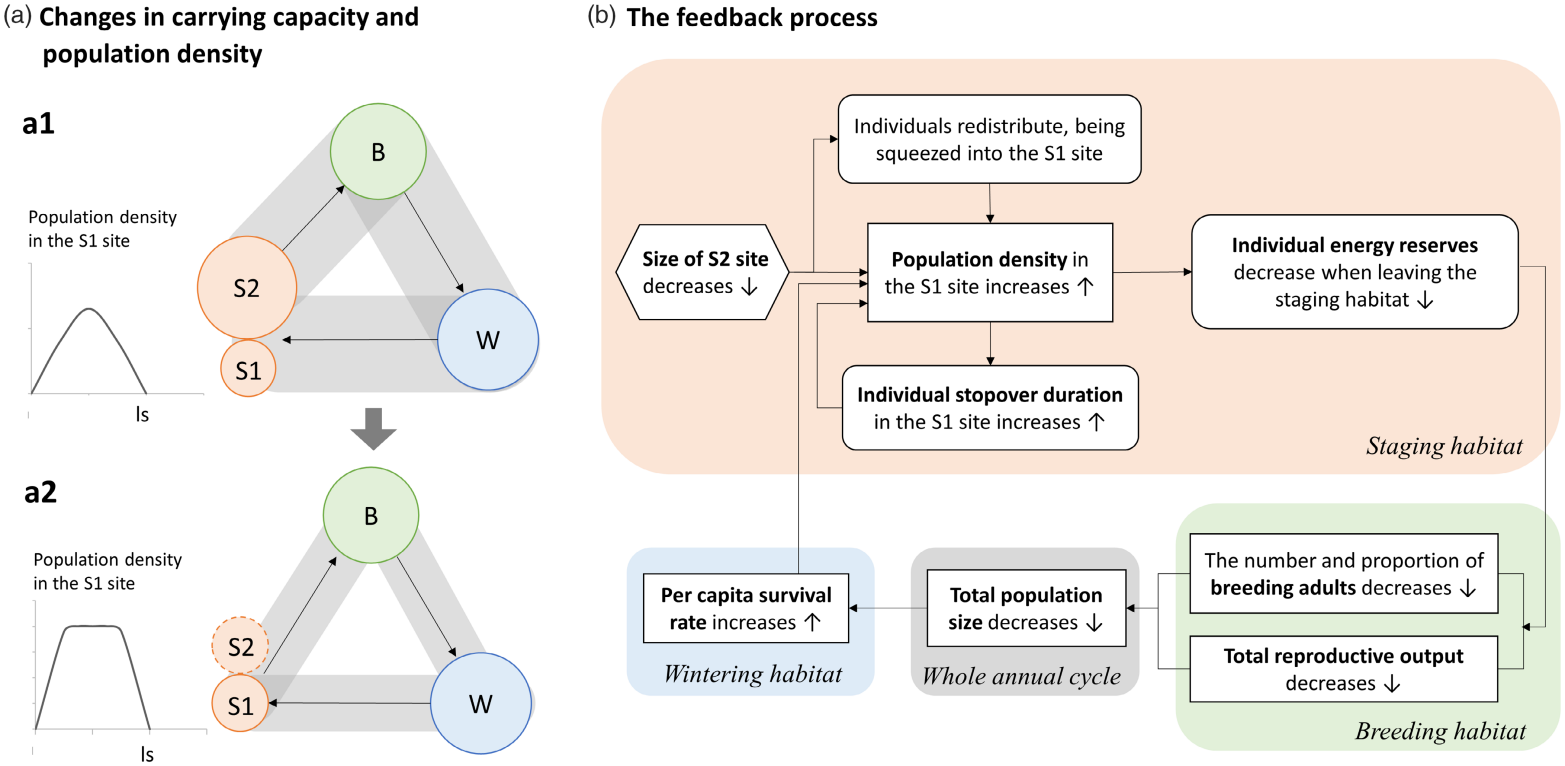
The processes linking individual migration tactics and population dynamics. (a) The changes in carrying capacity and population density as a function of the size of the staging habitat. The circle sizes represent the carrying capacity of each habitat, with the grey rectangles representing the maximum population size across the annual cycle. In (a1), the size of the wintering and breeding habitats determine carrying capacity, while in (a2) the size of the staging habitat determines it. The line charts show how population density in the remaining staging site changes during the period of migration stopover for scenarios (a1) and (a2) respectively. (b) The feedback process between individual stopover duration and population dynamics

By building a full‐annual‐cycle IBM of a stylized migratory population, we identify the critical role of stopover stage of northward migration in the influence of migration tactics and population dynamics of migratory birds across the whole annual cycle, also identify the key processes linking individual migration tactic and population dynamics (Figure 6). Our empirical data provides evidence to support the mechanisms shown from our theoretical model. Specifically, our results are consistent with the loss of staging habitat generating plasticity in migration tactic via increased intraspecific competition during migration stopovers. As the size of the staging area is reduced, individuals need to remain in the staging area for longer to obtain sufficient food to continue on their way due to an increase in the density of competitors. Our model shows that the consequence of this is individuals depart later, often with poor condition, and fewer individuals make it to the breeding area. However, those that do make it fare well. The dynamics at the staging area can consequently have knock‐on effects on populations in the overwintering and breeding areas that impact the population dynamics across the annual cycle, by altering the component of the life history where population dynamics are regulated.

Habitat loss in the staging habitat reduces the carrying capacity of the flyway, leading to population declines along it. As a consequence, the part of the annual cycle that determines the carrying capacity has been switched from the breeding habitat and wintering habitat to the staging habitat, as the spatial extent of the staging habitat decreases. The staging habitat during northward migration becomes the stage where the strongest density dependence operates, and the population becomes regulated by the carrying capacity of the staging habitat (Figure 6a). In contrast, competition in the breeding or wintering habitat is reduced, with processes operating in these areas no longer playing a major role in regulating the population dynamics. But any negative effects on the breeding or wintering grounds still can influence individual performance in the staging habitat through carry‐over effects (Norris, 2005; Ryan Norris & Marra, 2007). The contributions of different life‐history stages to the population dynamics can consequently vary with the spatial extent of the staging habitat. Such changes have the potential to alter selection on traits associated with competition for resources, and the entire life history.

Both in our simulations and empirical analysis, population density changes spatio‐temporally following habitat loss in the staging habitat, with a positive correlation between bird numbers and stopover duration (Figures 2b and 5b,d). This contrasts the ‘buffer effect’ hypothesis, which proposes that population density changes only in space (Brown, 1969; Gill et al., 2001; Sutherland, 1996a). Our results show that in addition to birds becoming more concentrated in the remaining area, a decrease in the extent of the staging area can also result in an increase in time spent there, leading to intensified competition during the staging period. Therefore, during the time‐limited northward migration, the reduced staging area not only leads to a higher population density in the staging habitat as individual birds stay longer, but also that the high population density is maintained for a longer period during this part of the life cycle (Figure 6a). And this can alter the strength of population regulation in either the wintering or breeding areas (Figure 6b). How do we reach these conclusions?

In our models, individuals adjust their migration tactic to prolong the stopover duration when food becomes scarce. Consequently, individuals often arrive at the breeding ground late and with low energy reserves, or they fail to reach the breeding grounds. This results in fewer adults arriving in the breeding ground, fewer adults reproducing and a decline in total reproductive output. However, those individuals that do arrive experience less competition and have higher departure energy reserves at the breeding habitat, breeding adults have higher per capita reproductive rate. All individuals have higher survival rate during southward migration and northward migration. Previous studies have reported evidence that support parts of the processes we describe here including: longer stopover duration is related to habitat loss, scarce food or high density of competitors (Conklin et al., 2021; Kelly et al., 2002; Moore & Yong, 1991), and decreased refuelling rate causes poor departure body mass in the staging area (Baker et al., 2004), limited refuelling time reducing survival rates in the northward migration (Rakhimberdiev et al., 2018) and reductions in total population‐level reproductive output (Desprez et al., 2018; Newton, 2006).

All of this is due to increased competition in the staging habitat, which reduces per capita food availability and generates behavioural plasticity in migratory tactics. As individual behaviour changes, so to do the population dynamics. The altered population dynamics, in turn, affect individual behaviour (Miner et al., 2005). This process continues until an equilibrium is reached, when the behaviour settles down to equilibrium, as do the population dynamics (Figure 6b). The connection between individual phenotypic plasticity and population dynamics is the result of feedback process across the annual cycle, which can result in eco‐evolutionary feedbacks are argued by Coulson (2021).

If the part of the annual cycle that determines carrying capacity is not operating in the staging habitat, individual tactics and population dynamics can show different patterns. The staging habitat was no longer the stage regulating population dynamics, and the strongest density‐dependent effects at the breeding or wintering habitat can cause a decrease in reproduction or survival rates at either stage, leading to a lower population density at the staging habitat, and a shortened stopover duration (Figure S6). Evidence has been reported by Holmes et al. (1996) and Rockwell et al. (2017)), who observed reduced survival rates when the wintering habitat is limiting, and Marra and Holmes (2001), Rodenhouse et al. (2003) and Tomotani et al. (2018)), who reported poor physical condition of juveniles and decreased reproduction when the breeding habitat is limiting. In addition, even though different breeding tactics may influence individuals' energy budgets across the annual cycle, individual stopover duration increased for both capital breeders and income breeders when facing the loss of staging habitats (Figure S6). Therefore, whether the changes in the extent of the staging habitat alters the part of the life cycle where carrying capacity is lowest will determine the direction of change in life‐history processes. Our proposed process can only occur when the staging habitat becomes the stage that determines the carrying capacity of the whole annual cycle.

Our empirical and theoretical results align well, and are consistent, with patterns reported in the existing literature on the EAAF migration flyway, which are decreasing trends in total population size along the flyway, increasing trends in population density (Clemens et al., 2016; Piersma et al., 2016; Studds et al., 2017; Wilson et al., 2011; Yang et al., 2011) and increasing stopover duration at the remaining staging area (Conklin et al., 2021). Our study reveals the underlying mechanisms behind a seemingly positive phenomena observed in migratory birds: the increasing counts are likely driven by longer stopover duration, combined with refugees squeezed from other staging sites that have declined in quality, rather than an overall population increase. Our study also highlights the crucial role of one life‐history stage to population dynamics across the whole annual cycle.

When constructing our theoretical model, we focused on the general mechanism caused by the loss of staging habitat, therefore we considered an average migratory tactic for all individuals, ignoring individual differences in migration behaviour driven by processes such as sex differences and a mixture distribution of migratory tactics. Males often migrate earlier than females in the northward migration to occupy the best breeding territories (Kokko et al., 2006; Newton, 2011), and some individuals might abort migration without making any breeding attempt (Lisovski et al., 2016; Shaw & Levin, 2011). However, as the lack of individual‐identified information on both timing, energy reserves and migrating trajectory, we chose to ignore them in this model. Nevertheless, individual differences in energy reserves persist throughout our model, and individuals with low energy reserves would not breed even they arrived at the breeding habitat. Therefore, individual differences might make our model more realistic, but are unlikely to change our main conclusions on the link between migratory tactics and population dynamics.

We call for further investigation on the connection between individual timing, energy reserves and demography across the whole annual cycle. Migratory birds need to be studied in depth at their breeding, wintering and staging areas to allow us to fully understand their dynamics. Focusing on a single area in detail, with limited data elsewhere, makes dynamical inference challenging, and may also lead researchers to reach erroneous conclusions, such as: the misinterpretation of increasing counts, and if the survey efforts were always put on the ‘best’ sites with increasing counts, this could lead to a situation where we keep seeing local increases in abundance right up until the point where species extinction is imminent, and lead us to miss the most opportune time for any conservation intervention. Migratory species play a key role for a number of other ecological processes by transporting energy and nutrients, or via their impacts on the ecological networks they form with other species in geographically separated areas (Bauer & Hoye, 2014), understanding their dynamics is also important to further extend our knowledge on their roles in ecosystem stability. Although logistically difficult, and costly, our work suggests that studies of migratory species across the entire annual cycle are necessary if we are going to understand the dynamics of species that exhibit one of the most remarkable behaviours in the animal kingdom.

## CONFLICT OF INTEREST

None of the authors have a conflict of interest.

## AUTHORS' CONTRIBUTIONS

J.L., T.C. and Z.Z. conceived the ideas and designed the methodology; J.L., W.L., X.M. and C.J.H. collected the data; J.L. built the model, analysed the data and led the writing of the manuscript with significant contributions from T.C. All authors contributed critically to the drafts and gave final approval for publication.

## Supporting information

Appendix S1‐S5

## Data Availability

Code and data are available at Dryad Digital Repository https://doi.org/10.5061/dryad.gxd2547p6 (Liu et al., 2022).
