# Supplementary material for: Unravelling the processes between phenotypic plasticity and population dynamics in migratory birds
Source: J Anim Ecol. 2022 Mar 23;91(5):983–95. doi: 10.1111/1365-2656.13686 (PMC9314967; doi:10.1111/1365-2656.13686)
Supplement: Supplementary file 1 — Appendix S1‐S5 [file JANE-91-983-s001.docx]

**Supporting Information**

for

**Unravelling processes between phenotypic plasticity and population dynamics in migratory birds**

Jin Liu, Weipan Lei, Xunqiang Mo, Chris J. Hassell, Zhengwang Zhang, Tim Coulson

**Appendix S1. Supplementary tables and figures**

Figure S1. The order of events and decisions in the IBM

Figure S2. Population dynamics predicted by model simulations.

Figure S3. Study area and pattern of habitat change in Bohai Bay between 2004 and 2018.

Figure S4. The approach of extracting stopover duration from the survey data

Figure S5. The proportion of individuals in different age classes and maturity status in three stages of the annual cycle.

Figure S6. Comparison of model outputs for different settings on the lowest carrying capacity.

Figure S7. Model outputs of varying 1% above the default parameter values.

Figure S8. The sensitivities of model outputs.

Figure S9. Temporal trends in waterbirds numbers of different survey sites; temporal trends in stopover duration of each common species.

Table S1. Different model settings on the lowest carrying capacity

Table S2. Parameters of the individual-based model

**Appendix S2. Supplementary information for the IBM**

**Appendix S3. Different model settings on the lowest carrying capacity**

**Appendix S4. Sensitivity analysis**

**Appendix S5. Supplementary information for empirical data**

S5.1 Study areas

S5.2 Data collection

S5.3 Identify common species

S5.4 Estimate stopover duration from the survey data

**Appendix S1. Supplementary figures and tables**


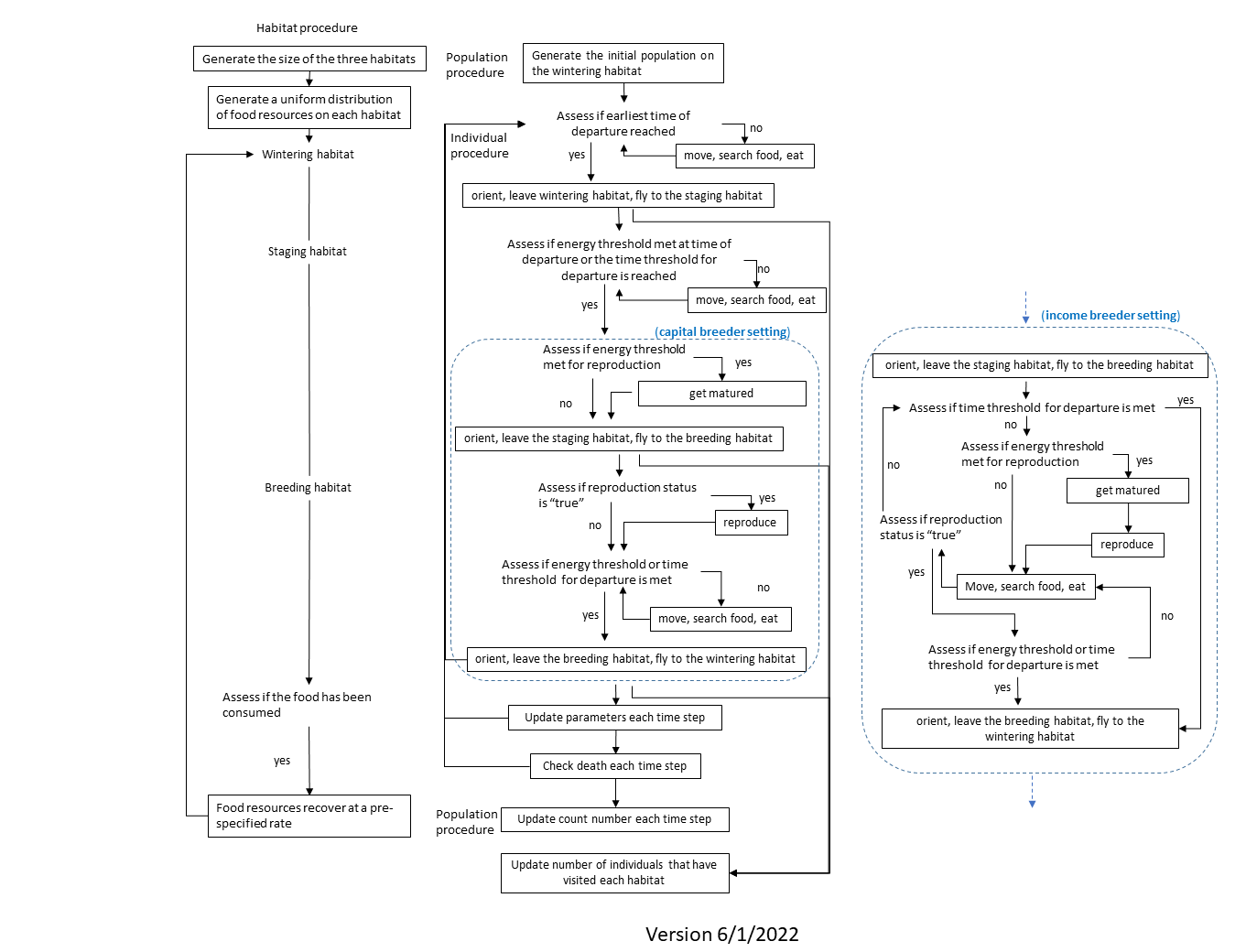


**Figure S1.** The order of events and decisions in the individual-based model of migrating birds. The events and decisions in the dashed boxes show different breeding tactics.


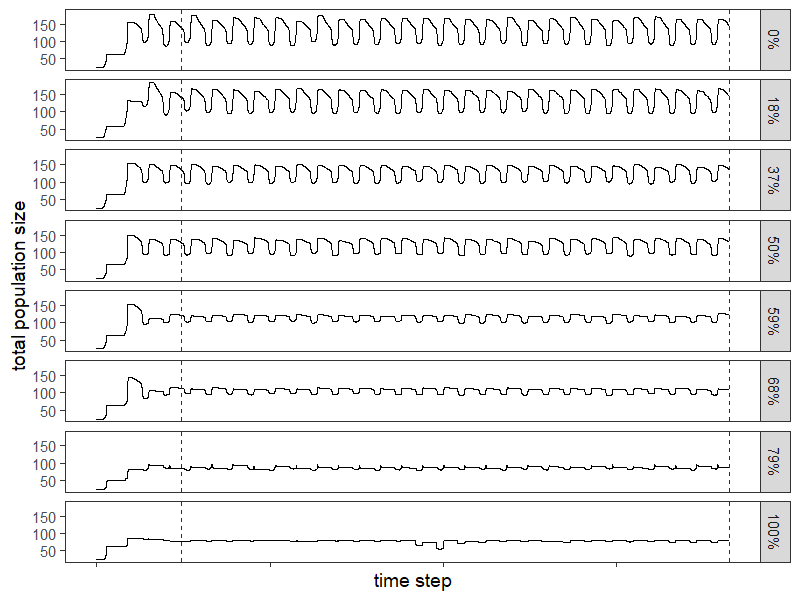


**Figure S2**. Population dynamics predicted by the model simulations. The facets are eight habitat scenarios in which the S2 site decreases from top to bottom, the numbers show the proportions of habitat loss of the S2 site. The black solid line represents the population size change over time in each scenario, which is the average from 10 runs. The two red dashed lines represent the start of year five and the end of year 30 respectively. The region between the two red dashed lines is where the population reaches stationary dynamics.


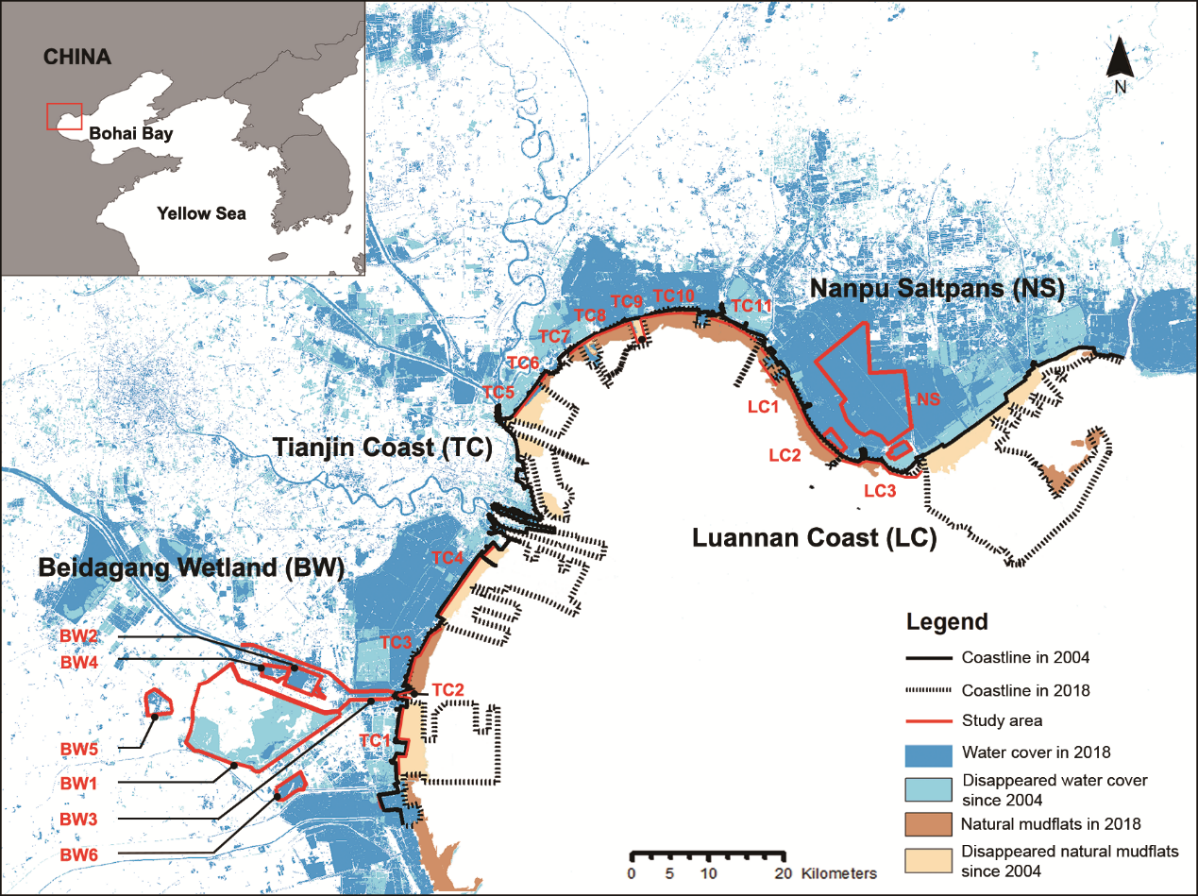


**Figure S3**. Study area and pattern of habitat change in Bohai Bay between 2004 and 2018. The study area is marked with red lines. The code for survey sites is denoted by the abbreviations of the four study regions, which are BW (Beidagang Wetland), TC (Tianjin Coast), LC (Luannan Coast) and NS (Nanpu Saltpans). Habitat change in water cover, mudflats and coastline are based on satellite images of Landsat 7 ETM SLC-off and Landsat 8 OLI_TIRS. See legend and text for details.


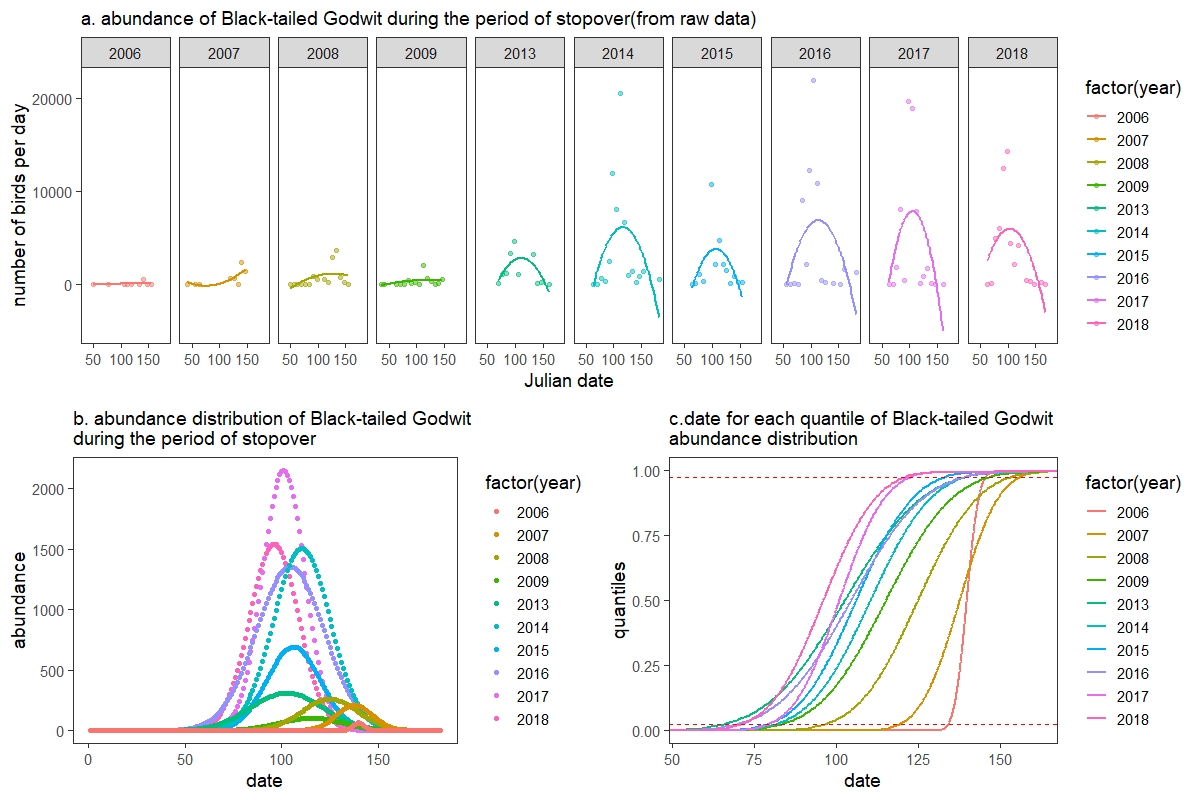


**Figure S4.** The approach of extracting stopover duration from survey data, taking Black-tailed Godwit (BTG) from the list of common species as an example. a) the abundance of BTG during the period of stopover each year from the survey data, fitted with curves of the quadratic function for Julian date. b) the abundance distribution curves of BTG during the period of stopover each year, which was estimated by the extracted mean date and variance in date from the survey data, and was scaled by bird number from the survey data. c) the quantile functions of the abundance distribution curves for each year, date at 2.5% quantile and 97.5% quantile is shown by dotted red lines.


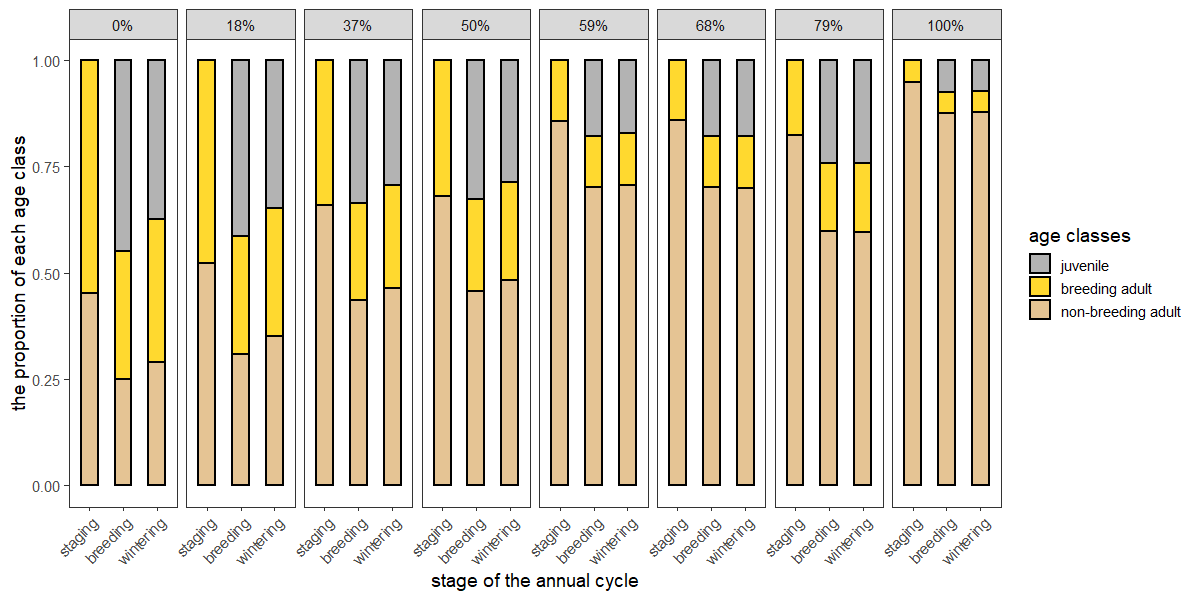


**Figure S5.** The proportion of individuals in different age classes and maturity status in three stages of the annual cycle across the eight habitat scenarios. The number on each facet shows the proportion of habitat loss of the S2 site.


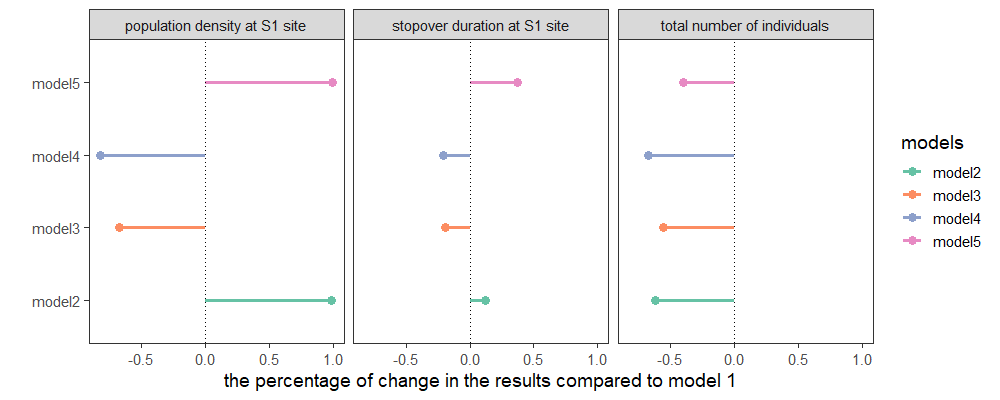


**Figure S6.** Comparison of model outputs for different settings on the lowest carrying capacity, including the average daily population density at the S1 site, the total number of individuals of the population and individual stopover duration at the S1 site. The distance from the point to the zero value line represents the percentage of change in the results of model 2-5 compared to model 1. Model 1 is the null model, with the same carrying capacity of all three habitats and a “capital breeding” tactic for reproduction; model 2 examined effects of the lowest carrying capacity at the staging habitat, with a “capital breeding” tactic for reproduction; model 3 examined effects of the lowest carrying capacity at the breeding habitat, with a “capital breeding” tactic for reproduction; model 4 examined effects of the lowest carrying capacity at the wintering habitat, with a “capital breeding” tactic for reproduction; model 5 examine the effects of breeding tactics, with the same carrying capacity setting with model 2, but with an “income breeding” tactic for reproduction.


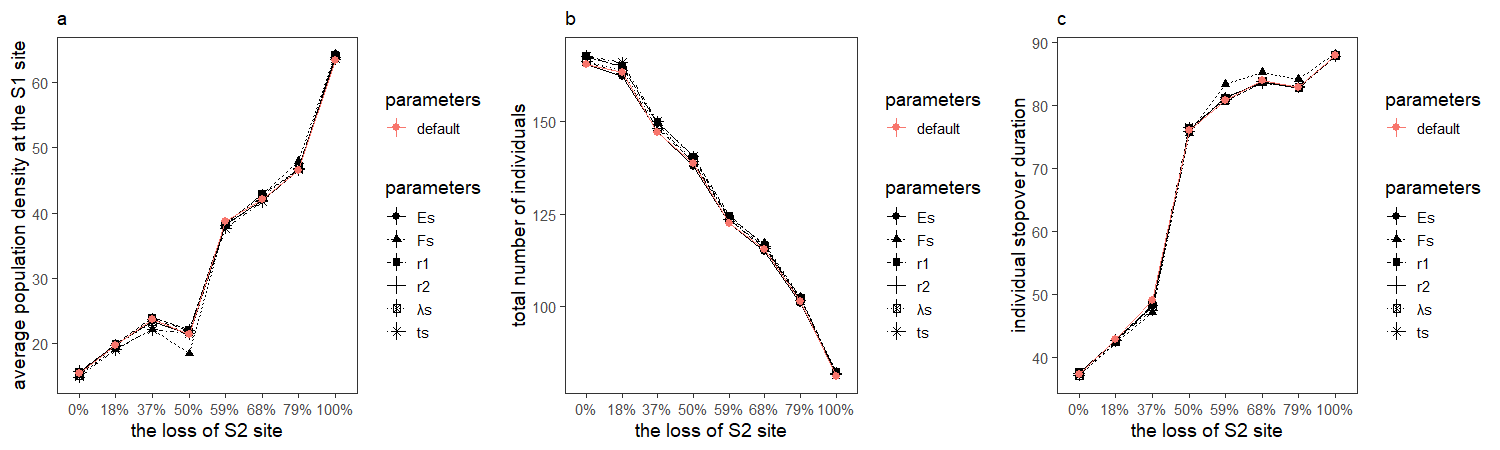


**Figure S7.** Model outputs across eight staging habitat scenarios by varying 1% above the default values of parameters. a) the average daily population density at the S1 site; b) the total number of individuals of the population; c) individual stopover duration at the S1 site. Parameters include: *F_s_* is the food density at the staging habitat, *λs* is the food recovery rate at the staging habitat, *r_1_* is the food-energy transformation rate at the staging habitat and breeding habitat, *r_2_* is the food-energy transformation rate at the wintering habitat, *E_s_* is the energy threshold for leaving the staging habitat, and *l_s_* is the time threshold for leaving the staging habitat.


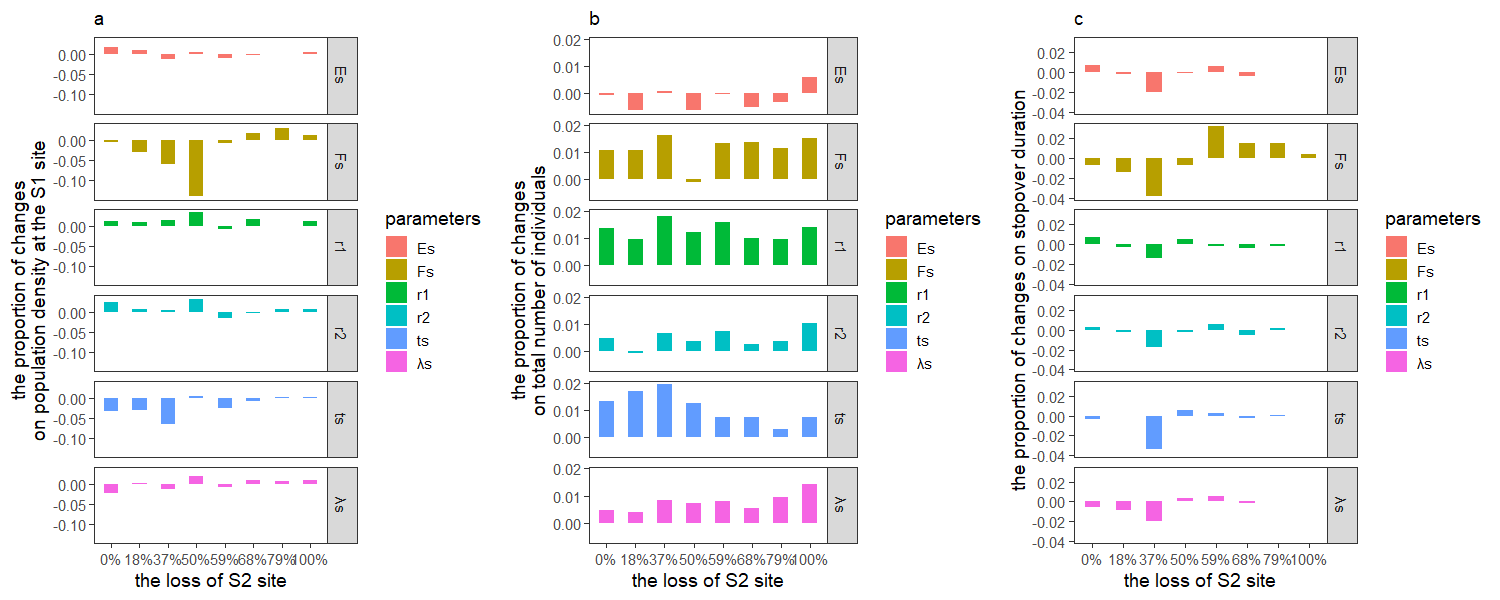


**Figure S8**. The sensitivities of model outputs to parameters for each habitat scenario: *F_s_* is the food density of the staging habitat, *λs* is the food recovery rate at the staging habitat, *r_1_* is the food-energy transformation rate at the staging habitat and breeding habitat, *r_2_* is the food-energy transformation rate at the wintering habitat, *E_s_* is the energy threshold for leaving the staging habitat, and *l_s_* is the time threshold for leaving the staging habitat.


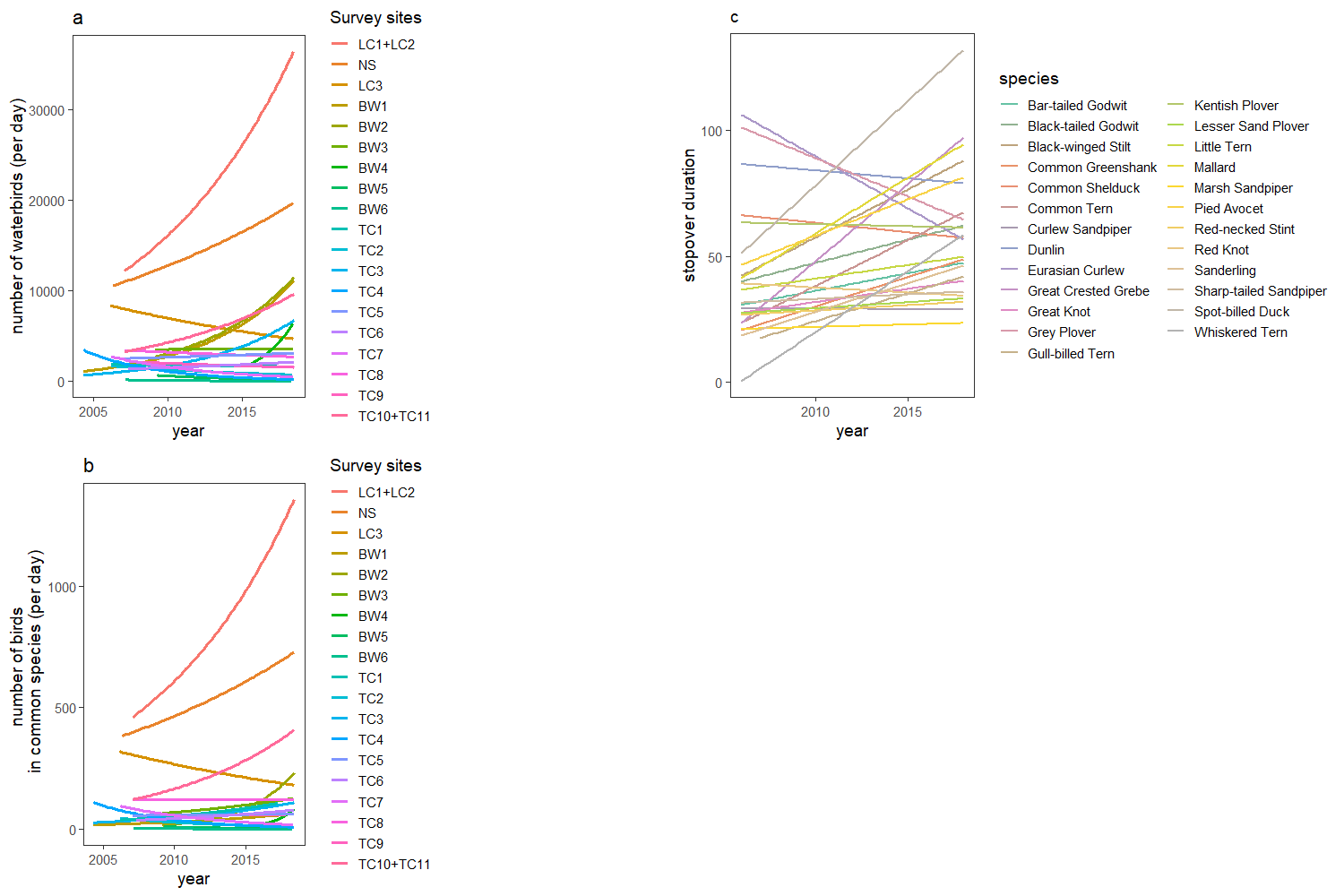


**Figure S9.** Temporal trends in waterbird abundance (a), the abundance of most common species (b) in different survey sites; temporal trends in stopover duration of each common species (c).

**Table S1.** Parameters of the individual-based models (see *Notes below for references of value settings)

| Parameters | Explanation | Value |
| --- | --- | --- |
| *N_0_* | Initial population size | 25 |
| *g* | The maximum age of individuals | 15 |
| *h* | the maximum number of female offspring | 2 |
| *F (F_s_,F_b_, F_w_)* | F_s_, Food density for each grid cell of the staging habitat (unit food)  F_b_, Food density for each grid cell of the breeding habitat (unit food)  F_w_, Food density for each grid cell of the wintering habitat (unit food) | 1 |
| *l_s_*^*^ | The latest departure day from the staging habitat (day of the year) | 150 |
|  | The latest departure day from the breeding habitat (day of the year) | 250 |
|  | The earliest departure day from the wintering habitat (day of the year) | 40 |
| *r_q_*^*^ (*r_1_, r_2_*) | *r_1_*, the food-energy transformation rate for the staging habitat and breeding habitat (unit energy/unit food) | 4.5 |
|  | *r_2_*, the food-energy transformation rate for the wintering habitat (unit energy/unit food) | 1.5 |
| *E_0_*^*^ | Initial energy reserve for adults (unit energy) | 100 |
|  | Initial energy reserve for offspring (unit energy) | 27 |
| *vf* | The speed of “fly” (grid cell/day) | 3 |
| *vm* | The speed of “move” (grid cell/day) | 1 |
| *ef_p, z_^*^* | The energy cost of behaviour “fly” of individual *p* at time *z* (unit energy) | 2.5 |
| *em_p, z_^*^* | The energy cost of behaviour “move” of individual *p* at time *z* (unit energy) | 1 |
| *eb_p, z_*^*^ | The energy cost of reproduction of individual *p* at time *z* (unit energy) | 80 |
| *E_s_*^*^ | The energy threshold for departure from the staging habitat (unit energy) | 180 |
|  | The energy threshold for departure from the breeding habitat (unit energy) | 150 |
| *E_sm_*^*^ | The energy threshold for reproduction (unit energy) | 180 |
| *λ* (*λ_s_, λ_b_, λ_w_*) | The rate of renewing food resources for each grid cell in the habitat after consumption | 0.5, 1 |
| *a_s_* | The sum size of S1 and S2 site (grid cell) | 306,267,226,198,  178,158,134,89 |
| *a_s1_* | The size of S1 (grid cell) | 89 |
| *a_s2_* | The size of S2 (grid cell) and the proportion of habitat loss in S2 site | 217 (0% loss),  178 (18% loss),  137 (37% loss),  109 (50% loss),  89 (59% loss),  56 (68% loss),  45 (79% loss),  0 (100% loss) |
| *a_b_* | Breeding habitat (grid cell) | 89, 178 |
| *a_w_* | Wintering habitat (grid cell) | 89, 178 |
| *n_x, z_* | The number of birds in grid cell *x* at time *z* | Calculated |
| *f_x, z_* | The amount of food in grid cell *x* at time *z* (unit food) | calculated |
| *e_p,q, z_* | The energy acquired by individual *p* at habitat *q* at time *z* (unit energy) | calculated |
| *E_p, z_* | The energy reserve of individual *p* at time *z* (unit energy) | calculated |
| *l^0^_p_* | The date that individual *p* arrived in the staging habitat | calculated |
| *l^1^_p_* | The date that individual *p* left the staging habitat | calculated |
| *L_p_* | Stopover duration of individual *p* in the staging habitat (days） | calculated |

*Notes:

Parameter values in this model were consensus values, drawn from empirical studies of multiple migratory waterbirds (Table S1), and values were not generated from statistical analyses, due to the lack of detailed individual-based data for both migration timing and energy budgets along the whole annual cycle for migratory birds.

1. *l_s_*. Studies of the migration timing for multiple species along the EAAF have shown that the day for birds leaving the wintering grounds is in the range of the 42^nd^ – 117^th^ day of the year, the day for birds leaving the staging area is in the range of the 79^th^ – 161^st^ day of the year, the day for birds leaving the breeding grounds is in the range of the 193^rd^ – 210^th^ day of the year (Lisovski *et al.* 2016a, Lisovski *et al.* 2016b, Chan *et al.* 2019, Lei *et al.* 2019, Lok *et al.* 2019, Kuang *et al.* 2020, Zhu *et al.* 2021). Since the tactic for migratory birds in the northward migration is usually time-minimized, and the tactic in the southward migration is usually energy-minimized (Hedenström and Alerstam 1997, Zhao *et al.* 2017), therefore the time threshold for the northward migration in our model was relatively tight on time, while the time threshold for the southward migration was relatively loose. Based on the information from existing literature, we set the earliest departure day from the wintering habitat in our model as the 40^th^ day of the year, the latest departure day from the staging habitat in our model is the 150^th^ day of the year, and the latest departure day from the breeding habitat in our model is the 250^th^ day of the year.
2. *r_q_*. The median accumulating rate of body mass at staging habitat is about 4-5g/day for a bird that weighs around 130g (Baker *et al.* 2004b). So, we set 4.5 unit energy /unit food as the food-energy transformation rate for the staging habitat and breeding habitat in our model. Since the body mass of migratory birds is usually maintained at a baseline level at wintering habitat, we set the food-energy transformation rate for the wintering habitat as 1.5 unit energy/unit food in our model (Piersma 2002, Zhao *et al.* 2016).
3. *Vf* and *vm*. Existing empirical studies show that the time spent in flight is often 10%-30% of the total time spent in northward migration for migratory waterbirds (Battley *et al.* 2012, Lisovski *et al.* 2016b, Conklin *et al.* 2021, Lisovski *et al.* 2021, Zhu *et al.* 2021). So we set the speed of “fly” was 3 grid cells per time step, to allow the ratio between the time spent in “fly” and the total time spent in the northward migration to fall in the range that empirical studies show. The speed of “move” was 1 grid cell per time step, to ensure individuals explore and compare the food values of every neighbouring grid cell at each time step to accumulate energy. The speeds have no impact on model predictions because the stopover duration is influenced by population density, food density and energy accumulations.
4. *ef_p, z_* and *ef_p, z_*. The energy expenditure during flight can be 2.5 times of basal metabolic rate for long-distance migrants, therefore we set the energy cost for “fly” was 2.5 times of the basic energy cost for “move” in our model (Alerstam and Lindström 1990, Hedenström and Alerstam 1998).
5. *E_s_*. The departure body mass is almost 1.5-1.8 times the arrival body mass for several migratory species at the staging habitat (Piersma and Jukema 1990, Ebbinge and Spaans 1995, Farmer and Wiens 1999, Baker *et al.* 2004a), so we set the energy threshold for departure here is 180 units energy.

For most migratory birds, the tactic for southward migration is usually energy cost minimized, so birds build up energy reserves for covering their migration distance but without overloading (Hedenström and Alerstam 1997). So we used the initial energy reserve set in our model plus the energy cost for covering the migration distance to estimate the departure energy threshold from the breeding habitat, which is 150 units of energy.

1. *eb_p, z_*. The cost for incubation is normally four times basal energy cost for breeding migrants (Piersma and Morrison 1994, Piersma 2002), as the basic energy cost for movement within the breeding habitat in our model is 1 unit energy/day, and the incubation duration is around 20 days for migratory waterbirds (Liebezeit *et al.* 2007, Kwon *et al.* 2018, Weiser *et al.* 2018), so we set the energy cost for reproduction in our model is 80 units energy in total (based on 4 ×1(unit energy/day) × 20 days).
2. *E_sm_*. After breeding, the body mass of females can drop to their lean mass (Ebbinge and Spaans 1995). So the energy threshold for reproduction in our model was 100(initial energy reserves) + 80(energy cost for reproduction) = 180 units of energy.
3. *E_0_*. Egg mass is estimated at 15% of female body mass for migratory shorebirds (Morrison and Hobson 2004). As in our model, breeding adults have 180 units energy reserves, so the initial energy reserves for offspring was set as 27 units energy.

| Models | a_s_^*^ (grid cell) | a_b_^*^(grid cell) | a_w_^*^(grid cell) | λ^*^ | Carrying capacity | Breeding tactics^*^ |
| --- | --- | --- | --- | --- | --- | --- |
| model 1 | 178 (S1-89, S2-89) | 178 | 178 | *λ_s_* = 1*, λ_b_* = 1*, λ_w_* =1 | The carrying capacity of the three types of habitats was the same | Capital breeding |
| model 2 | 89 | 178 | 178 | *λ_s_* = 0.5*, λ_b_* = 1*, λ_w_* =1 | The lowest carrying capacity occurred at staging habitat | Capital breeding |
| model 3 | 178 | 89 | 178 | *λ_s_* = 1*, λ_b_* = 0.5*, λ_w_* =1 | The lowest carrying capacity occurred at breeding habitat | Capital breeding |
| model 4 | 178 (S1-89, S2-89) | 178 | 89 | *λ_s_* = 1*, λ_b_* = 1*, λ_w_* =0.5 | The lowest carrying capacity occurred at wintering habitat | Capital breeding |
| model 5 | 89 | 178 | 178 | *λ_s_* = 0.5*, λ_b_* = 1*, λ_w_* =1 | The lowest carrying capacity occurred at staging habitat | Income breeding |

**Table S2** The parameters values under different model setting scenarios

***Notes**

1. *a_s_* is the sum size of S1 site and S2 site. *a_b_* is the size of the breeding habitat, *a_w_* is the size of wintering habitat, and *λ_s_* is the food recovery rate at the staging habitat, *λ_b_* is the food recovery rate at the breeding habitat, *λ_w_* is the food recovery rate at the wintering habitat.
2. Capital breeding models: Birds are capital breeders, they bring energy reserves with them to the breeding habitat, using the energy reserves stored from the staging habitat for reproduction. In the models, the procedure to assess the energy threshold for reproduction is performed prior to the procedure of leaving the staging habitat (Fig. S1).
3. Income breeding models: Birds are income breeders, they accumulate energy reserves after arriving at the breeding habitat, and use the energy stored from the breeding habitat for reproduction (Drent *et al.* 2006). In the models, the procedure to assess the energy threshold for reproduction is performed after arriving at the breeding habitat (Fig. S1).

**Appendix S2. Supplementary information of the IBM**

The model landscape was a two-dimensional space, consisting of 60*60 grid cells, conducted in NetLogo (Wilensky 1999). We allocated three types of habitats: the wintering habitat (W), the breeding habitat (B), and the staging habitat (S) split into two habitat types – S1 with 89 grid cells and S2. The size of S1 remained constant across all simulations, while the habitat size of S2 was adjusted to simulate the process of habitat loss in the staging habitat. The size of W and B was set as 178 grid cells. The total staging habitat was defined as the smallest rectangle that could encompass S1 and S2 such that individuals could move between S1 and S2 following the rules that defined movement in the staging habitat (see below). The size of the rectangle has no impact on model results, it is simply a device that allows individuals to move between S1 and S2 sites easily. Note that food was only available in S1 and S2 site, and not in the rest of the rectangle. Individuals only left the staging habitat to depart for the breeding habitat. The rest of the grid cells in the model landscape are non-habitat, where individuals can pass by during migration but do not stop.

Each grid cell within the breeding habitat, the wintering habitat, the S1 and S2 site contained renewing food resources which could grow to an upper limit, to provide a continuous source of energy for individuals, while the rest of the landscape (non-habitat, and the area other than S1 and S2 site of the total staging habitat) did not include any food resources. The value of food resources was uniformly distributed across the three habitats, and food resources at each grid cell of the habitat renewed each time step at the habitat-specified food recovery rate after consumption (Table S1). Each time step represented one day such that one year was comprised of 365 steps.

Each individual bird was characterised by individual identity, age, the status of reproduction (“true” – the individual is ready to reproduce in that year, or “false” – the individual is not ready to reproduce), and energy reserves. The age of individuals of the initial population was set to one, and increased by one at the end of overwintering each year. The maximum age of individuals was 15 years of our model. The initial status of reproduction was “false”.

In the model, the behaviours of each individual included: fly, move, search for food, eat, orient, mature, reproduce and die (Fig. S1). “Fly” was the movement across habitats during the course of migration. “Move” was the movement within each habitat during overwintering, staging and breeding. At the beginning of each simulation, birds were assigned by the model to the central grid cell of the wintering habitat. Birds flew between the three habitat types in the following order: wintering habitat to staging habitat, staging habitat to breeding habitat, and breeding habitat to wintering habitat. They flew towards the centre of the destination habitat. The centre of the breeding habitat and the wintering habitat was the central grid cell, the centre of the staging habitat was the midpoint of the line between the centres of the S1 site and the S2 site. Birds fly three grid cells per time step, with energy cost at each time step.

When birds reached each habitat, they started searching for food by following a random movement rule. They moved to the neighbouring grid cell with the highest food value by comparing the food value on their neighbouring eight grid cells to the current one. If there was a highest food value, birds moved towards this cell; if food values were the same in more than one cell, birds randomly chose a direction to move. Birds ate and increased their energy reserves when food was available, if food was unavailable, then birds randomly moved to a neighbouring cell and did not eat. There was no randomness of food acquisition, as long as the food resources were available, the behaviour of “eat” happened and energy was stored. To ensure that searching for food took place within the range of the habitat, boundaries between the habitat and non-habitat were established. Birds move one grid cell per time step with energy cost. When the energy reserves of an individual reached the energy threshold for departure, or the time reached for the latest possible departure arrived, the individual first oriented, adjusting its facing to the centre of the next destination habitat in the next time step, then flew towards it.

The order of “mature” procedure and “fly to the breeding habitat” procedure is different in the models with different breeding tactics (Fig S1). “mature” is the procedure that assessing whether ones energy reserves reached the energy threshold for reproduction, and changing one’s reproduction status if so. In the “capital breeding” models, when birds were at the staging habitat and their energy reserves reached the threshold for reproduction, they got matured and their reproduction status was set to “true” at the staging habitat. In the “capital breeding” models, the energy for reproduction relied on the stores from the staging habitat. In the “income breeding” models, the reproduction status was assessed at the breeding habitat, birds accumulated energy reserves when they did not reach the threshold for reproduction, when their energy reserves reached the threshold for reproduction, they got matured and their reproduction status was set to “true” at the breeding habitat. In the “income breeding” models, the energy for reproduction relied on stores from the breeding habitat.

When birds were at the breeding habitat, individuals with “true” values for reproduction status produce up to two offspring (either 1 or 2 was selected randomly), and the reproduction status changed to “false” after breeding. Each individual only reproduced once per year, and it lost energy through reproduction. Hatchlings hatched in the same grid cell as their parents, with initial age of zero, and an initial “false” value for reproduction status. Individuals aged 0 were categorized as juveniles, individuals aged 1 year or older with “true” value for reproduction were categorized as breeding adults, individuals aged 1 year or older with “false” value for reproduction were categorized as nonbreeding adults. Individuals aged 15 years, or with zero energy reserves, died and were removed from the population.

**Energy reserve**

The energy reserve of each individual was assumed to be dependent on their initial energy, energy gained, and energy expended. The expected energy gained from food relied on both population density and food density (Goss-Custard *et al.* 2002), and there was no randomness of food acquisition, as :

$e_{p, q,z}= \frac{f_{x, z}}{n_{x, z}}\times r_{q}- {em}_{p, z}-{ef}_{p, z}-{eb}_{p,z}$ (A1)

$E_{p,z+1}=E_{p, z}+e_{p, q,z}$ (A2)

where *e_p,q,z_* is the energy acquired by individual *p* at habitat *q* at time *z*, *f _x,z_* is the amount of food in grid cell *x* at time *z*, *n_x,z_* is the number of birds in grid cell *x* at time *z*, *r_q_* is the food-energy transformation rate at habitat *q*, *em_p,z_* is the energy cost of “move”, *ef_p,z_* is the energy cost of “fly”, *eb_p,z_* is the energy cost of reproduction, and *E_p,z_* is the total energy reserve of individual *p* at time *z*. All parameters were the same for all individuals regardless of age.

**Stopover duration in staging habitat**

The stopover duration of a bird in the staging habitat was related to the energy requirement for migration, and the rate of energy acquisition (Hedenström and Alerstam 1997). In our model, the conditions for leaving the staging habitat were either the energy reserves exceeded the energy threshold (A3) or time passed the time threshold (A4), as:

$E_{p,z}>E_{s}$ (A3)

$z=l_{s}$ (A4)

where *E_p,z_* is the energy reserves of individual *p* at time step *z*, *E_s_* is the energy threshold for departure from the staging habitat, and *l_s_* is the time threshold for departure from the staging habitat.

$L_{p}=l_{p}^{1}-l_{p}^{0}$ (A5)

where *L_p_* is the stopover duration for individual *p*, and *l^0^_p_* is the time step when individual *p* arrived at the staging habitat, *l^1^_p_* is the time step when individual *p* left the staging habitat.

**Appendix S3. The comparison between different model settings**

To further examine whether our hypothesized processes only occur when the staging habitat became the stage with the lowest carrying capacity along the annual cycle, we tested impacts on individual stopover duration and population dynamics by reducing carrying capacity at other life cycle stages. Five types of models were constructed in our study, with models differing in the size of the S2 site, the size of the breeding habitat and the size of the wintering habitat, the food recovery rate in each of these three habitats, and the breeding tactics individuals followed (Table S2). More specifically:

- Model 1 was the null model, with the same size and the food recovery rate at all three habitats, and a “capital breeding” tactic for reproduction.
- Model 2 examined the effects of the size of the staging habitat on individual tactics and population dynamics, with eight scenarios where the size of S2 area was varied were run, with the lowest food recovery rate at the staging habitat and a “capital breeding” tactic for reproduction.
- Model 3 examined effects of the lowest carrying capacity at the breeding habitat to individual tactics and population dynamics, with the lowest size and food recovery rate at the breeding habitat and a “capital breeding” tactic for reproduction.
- Model 4 examined effects of the lowest carrying capacity at the wintering habitat to individual tactics and population dynamics, with the lowest size and food recovery rate at the wintering habitat and a “capital breeding” tactic for reproduction.
- Model 5 examine the effects of breeding tactics to individual stopover tactics and population dynamics, with the same settings of habitat size and food recovery rate with model 2, but with an “income breeding” tactic for reproduction.

By comparing the results from models (model 2, 3, 4) with the lowest carrying capacity at different life cycle stages and the results from the null model (model 1), we found our hypothesized processes only occur when the lowest carrying capacity was at the staging habitat. The processes are: population density at the S1 site increased, individuals remained longer at the staging habitat, with lower energy reserves when leaving the staging habitat, leading to fewer breeding adults and juveniles in the population and lower per capita reproduction rate. Individuals that bred had better performance compared to other models, with higher energy reserves when leaving the breeding and wintering habitat, and higher survival rate during migration. However, when the lowest carrying capacity was at other life cycle stages (model 3 - breeding stage or model 4 - wintering stage), although the trends of the total number of individuals decreased as the same, the processes were completely opposite, with decreased population density at the S1 site and shortened individual stopover duration. By comparing the results from models with different breeding tactic settings (model 2 – capital breeding, model 5 – income breeding), we found different breeding tactics did not influence the processes linking migration tactic and population dynamics across the life cycle (Fig S6).

**Appendix S4. Sensitivity analysis**

**S4.1 Methods**

We focused on three types of output: average daily population density at the S1 site, which was denoted as *N_d_*; the total number of individuals within the stopover period at the staging habitat, which was denoted as *N_e_*; and individual stopover duration *L*, we calculated the sensitivity coefficients of each model output to each parameter respectively as:

$\frac{\partial_{\gamma, a_{s}}}{\partial_{\theta}}, \gamma=N_{d}, N_{e}, L; \theta=F_{s}, \lambda_{s}, r_{1}, r_{2},E_{s},l_{s}$ (A6)

where *γ* is the type of model output we focused on (including *N_d_*, *N_e_* and *L*), and *θ* represents the parameters we tested (including *F_s_*, *λ_s_*, *r_1_*, *r_2_*, *E_s_*, and *l_s_* as shown in Table S2), *a_s_* is the size of staging habitat as shown in Table S2.

We varied parameter values to 101% of the default values (shown in Table S2), and estimated the mean of the outputs in the default and 101% parameter value for eight habitat scenarios respectively. $\partial_{\gamma, a_{s}}$ is the difference between the mean of the outputs in 101% parameter values and the mean of the outputs in the default values at each habitat scenario. (Railsback and Grimm 2019).

**S4.2 Results**

The trends of model outputs through eight habitat scenarios were robust (Fig S7), although the magnitude of the effects on three main outputs differed between habitat scenarios (Fig S8). Population density at the S1 site and individual stopover duration was most affected by the time threshold for departure and food density. The increase of *t_s_* reduced the population density and individual stopover duration, suggesting *t_s_* release the competition for food at the S1 site, due to the population having a longer period. The increase of *F_s_* decreased the population density and individual stopover duration when the loss of S2 was less than 50% but increased these two outputs when the loss of S2 was higher than 50%. It suggests higher food density reduces the competition when the alternative staging site was large in size, but increases the competition when the alternative staging site was small. The total number of individuals was positively affected by *F_s_*, *r_1_* and *t_s_*. The increase of *F_s_* increased the carrying capacity, therefore leading to more individuals surviving in the population. *r_1_* had the strongest effects on the total number of individuals, and the increase of *r_1_* increased the energy accumulating rates, causing individuals to reach the energy threshold faster. The increase of *t_s_* released the competition at the S1 site leading more individuals to survive. Stopover duration was most affected by food density, with the time threshold following.

Food density, the time threshold and the food-energy transformation rate at staging habitats had the most impacts on the model outputs, and the degree of impacts varies with the size of the staging habitat. Although the value of model outputs changed when manipulating the parameters, the trends of model outputs through eight habitat scenarios did not change.

**Appendix S5. Supplementary information of empirical data**

**S5.1 Study areas**

Our empirical study was conducted in the wetlands in the north of Bohai Bay, between 38°36’-39°13’N and 117°11’-118.22’E, located in the northwest of the Yellow Sea (Fig. S3), an area which is 102 km from west to east and 70 km from north to south. Bohai Bay is characterized by the typical continental climate, with significant winds, cold dry winters and hot humid summers. The annual mean temperature is 10.7℃ and the mean annual precipitation is 500-600mm. Ice formation begins in December and ends by March (Editorial Committee of Encyclopedia of China 1992). The coastal region is flat and low-lying and 135km long. The intertidal mudflats range from 1-3 km in width at low tide, and would be completely submerged from about two hours before high tide. There are extensive saltpans, shrimp ponds, fish ponds and reservoirs along the coast, several large rivers flowing into Bohai Bay (Barter *et al.* 2003, Yang *et al.* 2011), and also a large area of reclaimed land for harbours, wharves, oil fields, and industrial factories.

We focused on four regions in this area (Fig.S3): (1) Beidagang Wetland of Tianjin (38°36'-38°50' N, 117°11'-117°34' E) is a nature reserve covering about 348.87 km^2^, consisting of rivers, marsh, grassland, reservoirs and ponds. Waterbirds make use of this region for feeding and roosting. (2) Tianjin Coast (38°45'-39°13'N, 117°34'-118°1'E) and (3) Luannan Coast (39°0'-39°12'N, 118°3'-118°22'E) which together cover 131.65 km of coastline, comprising tidal mudflats and reclamation areas. Waterbirds (mainly shorebirds) make use of tidal mudflats in these regions for foraging. (4) Nanpu Saltpans (39°1'-39°12', 118°8'-118°20'E) covers an area of 290 km^2^, consisting of shallow, interconnected pans separated by dykes, shrimp ponds and fish ponds. Waterbirds use this area for both foraging and roosting, especially during the high tide (Lei *et al.* 2018).

**S5.2 Data collection**

We used 13 years of survey data of waterbirds collected between 2004 and 2018 in the north of Bohai Bay. Data were collected in all years except for 2005 and 2011, when the fieldwork was not fully carried out. In this study, we focus on the northward migration from February to June. Surveys were conducted at least twice a month throughout the migration season.

A total of 148 waterbird species were recorded and analysed from 811 observation days between 2004 and 2018. All waterbirds were identified to species when possible within each survey. The number of waterbirds by species was recorded, while unidentifiable species that were partially obscured by vegetation or distance from observers were recorded as “unidentified”. Those “unidentified” species were included in analyses when examining the total abundance of waterbirds but were not used in other analyses. In addition, the date, the survey site name, and the number of observers for each survey were also recorded in the dataset to correct bias caused by observers and survey range.

Surveys covered 285.36 km^2^ area of Beidagang Wetland, 104.01 km coastline of Tianjin coast, 20 km of coastline of Luannan coast and 93 km^2^ area of Nanpu saltpans (Fig.S7). Beidagang Wetland and Tianjin coast were surveyed at each data collection, while Luannan coast was not surveyed in 2004, 2010, 2012 and 2013. Similarly, Nanpu saltpans were not surveyed in 2004, 2009, 2010 and 2012. Within the four regions, 23 survey sites were monitored. The time coverage of these survey sites was varying from 7.7% to 100%. Nineteen survey sites were included in the analysis, with four survey sites excluded because they had <30% time coverage.

Each survey followed the same objectives: to count all waterbirds and to avoid double-counting in each survey location. Waterbirds were counted with telescopes (25-60×magnification eyepieces) and binoculars. To survey waterbirds in Beidagang Wetland and Nanpu saltpans, counts were conducted from survey points on the roads or levees, while surveys in coastal areas were conducted from points on dykes close to the sea. We used obvious geographical indicators (such as a pond, vegetation boundaries, buildings, fishing nets, piles of debris and rocks) to minimize the likelihood of double counting.

Some waterbirds forage on the tidal flat at low tide and roost in saltpans at high tide (Lei *et al.* 2018). To avoid double-counting, observers visited sites in Nanpu saltpans synchronously with the adjacent coastal sites during low tide in all surveys except for 2013 and 2015. In 2013 and two surveys in 2015, the saltpans were visited independently during high tide when coastal sites had been submerged. Sites in Beidagang wetland were visited just before or after the counts in coastal sites, where waterbird movement is independent of the tidal cycle.

**S5.3 common species**

The “Frequency Based Method” focused on the frequency of each species *i,* ($f_{i}$) , by using the relative abundance (Preston 1948, Magurran and Henderson 2011). We calculated the frequency of each species within the whole community each day, and obtained the average value for each species over all the survey days as:

$f_{i}=\frac{\sum_{j}^{J} \frac{n_{i,j}}{N_{j}}}{J}$ (A7)

Where *i* is species, *J* is the total number of observation days, *n_i,j_* is the population size of species *i* on day *j*, and *N*_j_ is the total number of waterbirds counted on day *j*. The “Distribution Based Method” focused on the species distribution. We used the number of occupied sites to define commonness (Pearman & Weber 2007) and ranked all species by averaging the number of sites occupied by each species *i* each year *y*, which is denoted as *K_i,y_*. By setting *f_i_* >= 0.005 and *K_i,y_* >= 3, excluding gulls from the list (since seabirds have different migration patterns compared to other waterbirds), 25 most common species were identified.

The most common species were: Bar-tailed Godwit(*Limosa lapponica*), Black-tailed Godwit (*Limosa limosa*), Black-winged Stilt (*Himantopus himantopus*), Common Greenshank *(Tadorna tadorna*), Common Shelduck (*Tadorna tadorna*), Common Tern (*Sterna hirundo*), Curlew Sandpiper (*Calidris ferruginea*), Dunlin *(Calidris alpina*), Eurasian Curlew (*Numenius arquata*), Great Crested Grebe (*Podiceps cristatus*), Great Knot (*Calidris tenuirostris*), Grey Plover (*Pluvialis squatarola*), Gull-billed Tern (*Gelochelidon nilotica*), Kentish Plover (*Charadrius alexandrinus*), Lesser Sand Plover (*Charadrius mongolus*), Little Tern (*Sternula albifrons* ), Mallard (*Anas platyrhynchos*), Marsh Sandpiper (*Tringa stagnatilis*), Pied Avocet (*Recurvirostra avosetta*), Red-necked Stint (*Calidris ruficollis*), Red Knot *(Calidris canutus)*, Sanderling (*Calidris alba*), Sharp-tailed Sandpiper (*Calidris acuminata*), Spot-billed Duck (*Anas zonorhyncha* ), Whiskered Tern (*Chlidonias hybrida*).

**S5.4 Estimate stopover duration from the survey data**

Since the quadratic relationship between Julian date and bird abundance was revealed from our survey data (Fig. S4 a), we first estimated normal distributions of bird abundance within the period of stopover for each species each year (Fig. S4b). To do this, we extracted the mean date and variance in date for each species each year from survey data, and scaled the curves by the bird number from the survey data. Then we estimated the date by which each quantile of the distribution of bird abundance is reached, date at 2.5% quantile and 97.5% quantile was the date of arrival at staging habitat and the date of departure from staging habitat for each species each year within 95% confidential interval respectively (Fig. S4c).

Since the survey dates were different each year, in order to build comparable curves between years, we calculated the mean abundance of each species *i* each week *w* each year *y*, denoting it as *N_i,w,y_*, used the Julian date of the middle date of each week to represent the date for the abundance, denoting it as *tm_i,w,y_*. Year 2004, 2005, 2010, 2011, 2012 were excluded from this analysis, due to lack of sufficient data for extracting stopover duration. We calculated the mean date within the stopover period for each species each year (*μ_i,y_*), and the variance of the date in the stopover period for each species each year ($\delta_{i,y}$) by:

$\mu_{i,y}=\frac{\sum({tm}_{i,w,y}*N_{i,w,y})}{\sum N_{i,w,y}}$ (A8)

$\delta_{i,y}= \sqrt{\frac{\sum{{(tm}_{i,w,y}}^{2}{*N}_{i,w,y})}{\sum N_{i,w,y}}}-{\mu_{i,y}}^{2}$ (A9)

We estimated the abundance by the probability density function N(*μ_i,y_,* ${\delta_{i,y}}^{2}$) in the range of day 1 to day 182 by using dnorm() function in the program R. Then we estimated the quantile functions by using qnorm() in the program R, date at 2.5% quantile and date at 97.5% quantile was the arrival date ($t_{i,y}^{0}$) and departure date ($t_{i,y}^{1}$) within 95% confidential interval respectively. We calculated the stopover duration for each species each year (*T_i,y_)* by:

*T_i,y_* = $t_{i,y}^{1}- t_{i,y}^{0}$ （A10）

**Appendix references**

Alerstam, T., and Å. Lindström. 1990. Optimal bird migration: the relative importance of time, energy, and safety. Pages 331-351 Bird migration. Springer.

Baker, A. J., P. M. Gonzalez, T. Piersma, L. J. Niles, I. de Lima Serrano do Nascimento, P. W. Atkinson, N. A. Clark, C. D. Minton, M. K. Peck, and G. Aarts. 2004a. Rapid population decline in red knots: fitness consequences of decreased refuelling rates and late arrival in Delaware Bay. *Proceedings of the Royal Society of London. Series B: Biological Sciences* **271**:875-882.

Baker, A. J., P. M. González, T. Piersma, L. J. Niles, I. de Lima Serrano do Nascimento, P. W. Atkinson, N. A. Clark, C. D. T. Minton, M. K. Peck, and G. Aarts. 2004b. Rapid population decline in red knots: fitness consequences of decreased refuelling rates and late arrival in Delaware Bay. *Proceedings of the Royal Society of London. Series B: Biological Sciences* **271**:875-882.

Barter, M. A., A. Riegen, and Q. Xu. 2003. Shorebird numbers in Bohai Wan during northward migration. *The Stilt* **44**:3-8.

Battley, P. F., N. Warnock, T. L. Tibbitts, R. E. Gill Jr, T. Piersma, C. J. Hassell, D. C. Douglas, D. M. Mulcahy, B. D. Gartrell, and R. Schuckard. 2012. Contrasting extreme long‐distance migration patterns in bar‐tailed godwits Limosa lapponica. *Journal of Avian Biology* **43**:21-32.

Chan, Y. C., T. L. Tibbitts, T. Lok, C. J. Hassell, H. B. Peng, Z. Ma, Z. Zhang, and T. Piersma. 2019. Filling knowledge gaps in a threatened shorebird flyway through satellite tracking. *Journal of Applied Ecology* **56**:2305-2315.

Conklin, J. R., S. Lisovski, and P. F. Battley. 2021. Advancement in long-distance bird migration through individual plasticity in departure. *Nature Communications* **12**:1-9.

Drent, R. H., A. D. Fox, and J. Stahl. 2006. Travelling to breed. *Journal of Ornithology* **147**:122-134.

Ebbinge, B. S., and B. Spaans. 1995. The importance of body reserves accumulated in spring staging areas in the temperate zone for breeding in dark-bellied brent geese Branta b. bernicla in the high Arctic. *Journal of Avian Biology*:105-113.

Editorial Committee of Encyclopedia of China. 1992. Encyclopedia of China, Chinese geography. Encyclopedia of China Publishing House, Beijing, China.

Farmer, A. H., and J. A. Wiens. 1999. Models and reality: Time–energy trade‐offs in pectoral sandpiper (calidris melanotos) migration. *Ecology* **80**:2566-2580.

Goss-Custard, J. D., R. A. Stillman, A. D. West, R. W. G. Caldow, and S. McGrorty. 2002. Carrying capacity in overwintering migratory birds. *Biological conservation* **105**:27-41.

Hedenström, A., and T. Alerstam. 1997. Optimum fuel loads in migratory birds: distinguishing between time and energy minimization. *Journal of theoretical biology* **189**:227-234.

Hedenström, A., and T. Alerstam. 1998. How fast can birds migrate? *Journal of Avian Biology*:424-432.

Kuang, F., J. T. Coleman, C. J. Hassell, K.-S. K. Leung, G. Maglio, W. Ke, C. Cheng, J. Zhao, Z. Zhang, and Z. Ma. 2020. Seasonal and population differences in migration of Whimbrels in the East Asian–Australasian Flyway. *Avian Research* **11**:1-12.

Kwon, E., W. B. English, E. L. Weiser, S. E. Franks, D. J. Hodkinson, D. B. Lank, and B. K. Sandercock. 2018. Delayed egg‐laying and shortened incubation duration of Arctic‐breeding shorebirds coincide with climate cooling. *Ecology and Evolution*

**8**:1339-1351.

Lei, J., Y. Jia, A. Zuo, Q. Zeng, L. Shi, Y. Zhou, H. Zhang, C. Lu, G. Lei, and L. Wen. 2019. Bird satellite tracking revealed critical protection gaps in East Asian–Australasian Flyway. *International journal of environmental research* **16**:1147.

Lei, W., J. A. Masero, T. Piersma, B. Zhu, H.-Y. Yang, and Z. Zhang. 2018. Alternative habitat: the importance of the Nanpu Saltpans for migratory waterbirds in the Chinese Yellow Sea. *Bird Conservation International* **28**:549-566.

Liebezeit, J. R., P. A. Smith, R. B. Lanctot, H. Schekkerman, I. Tulp, S. J. Kendall, D. M. Tracy, R. J. Rodrigues, H. Meltofte, and J. A. Robinson. 2007. Assessing the development of shorebird eggs using the flotation method: species-specific and generalized regression models. *The Condor* **109**:32-47.

Lisovski, S., K. Gosbell, M. Christie, B. J. Hoye, M. Klaassen, I. D. Stewart, A. J. Taysom, and C. Minton. 2016a. Movement patterns of sanderling (Calidris alba) in the East Asian-Australasian Flyway and a comparison of methods for identification of crucial areas for conservation. *Emu-Austral Ornithology* **116**:168-177.

Lisovski, S., K. Gosbell, C. Hassell, and C. Minton. 2016b. Tracking the full annual-cycle of the Great Knot, Calidris tenuirostris, a long-distance migratory shorebird of the East Asian-Australasian Flyway. *Wader Study* **123**.

Lisovski, S., K. Gosbell, C. Minton, and M. Klaassen. 2021. Migration strategy as an indicator of resilience to change in two shorebird species with contrasting population trajectories. *Journal of Animal Ecology* **90**:2005-2014.

Lok, T., C. J. Hassell, T. Piersma, R. Pradel, and O. Gimenez. 2019. Accounting for heterogeneity when estimating stopover duration, timing and population size of red knots along the Luannan Coast of Bohai Bay, China. *Ecology and Evolution* **9**:6176-6188.

Magurran, A. E., and P. A. Henderson. 2011. Commonness and rarity. *Biological Diversity: Frontiers in Measurement Assessment*:97-104.

Morrison, R. I. G., and K. A. Hobson. 2004. Use of body stores in shorebirds after arrival on high-arctic breeding grounds. *The Auk* **121**:333-344.

Piersma, T. 2002. Energetic bottlenecks and other design constraints in avian annual cycles. *Integrative and comparative biology* **42**:51-67.

Piersma, T., and J. Jukema. 1990. Budgeting the flight of a long-distance migrant: changes in nutrient reserve levels of bar-tailed godwits at successive spring staging sites. *Ardea* **55**:315-337.

Piersma, T., and R. G. Morrison. 1994. Energy expenditure and water turnover of incubating ruddy turnstones: high costs under high arctic climatic conditions. *The Auk* **111**:366-376.

Preston, F. W. 1948. The commonness, and rarity, of species. *Ecology* **29**:254-283.

Railsback, S. F., and V. Grimm. 2019. Agent-based and individual-based modeling: a practical introduction. Princeton University Press.

Weiser, E. L., S. C. Brown, R. B. Lanctot, H. R. Gates, K. F. Abraham, R. L. Bentzen, J. Bêty, M. L. Boldenow, R. W. Brook, and T. F. Donnelly. 2018. Effects of environmental conditions on reproductive effort and nest success of Arctic‐breeding shorebirds. *Ibis* **160**:608-623.

Wilensky, U. 1999. NetLogo. Center for Connected Learning and Computer-Based Modeling, Northwestern University. Evanston, IL.

Yang, H.-Y., B. Chen, M. Barter, T. Piersma, C.-F. Zhou, F.-S. Li, and Z.-W. Zhang. 2011. Impacts of tidal land reclamation in Bohai Bay, China: ongoing losses of critical Yellow Sea waterbird staging and wintering sites. *Bird Conservation International* **21**:241-259.

Zhao, M., R. Atkinson, M. Bennett, M. Christie, K. Gosbell, B. Hoye, P. Johns, S. Lisovski, C. Minton, R. J. C. Patrick, and s. o. l.-d. m. s. a. t. E. A.-A. Flyway. 2016. Constraints strategies of long-distance migratory shorebirds along the East Asian-Australasian Flyway. *PhD thesis*:65.

Zhao, M., M. Christie, J. Coleman, C. Hassell, K. Gosbell, S. Lisovski, C. Minton, and M. Klaassen. 2017. Time versus energy minimization migration strategy varies with body size and season in long-distance migratory shorebirds. *Movement Ecology* **5**:23.

Zhu, B.-R., M. A. Verhoeven, A. J. Loonstra, L. Sanchez-Aguilar, C. J. Hassell, K. K. Leung, W. Lei, Z. Zhang, and T. Piersma. 2021. Identification of breeding grounds and annual routines of the newly discovered bohaii subspecies of Black-tailed Godwits. *Emu-Austral Ornithology* **121**:292-302.
